# Supplementary material for: Fair algorithms for selecting citizens’ assemblies
Source: Nature. 2021 Aug 4;596(7873):548–52. doi: 10.1038/s41586-021-03788-6 (PMC8387237; doi:10.1038/s41586-021-03788-6)
Supplement: Supplementary file 1 — This file contains Supplementary Sections 1-15 – see Contents page for details. [file 41586_2021_3788_MOESM1_ESM.pdf]

nature portfolio

https://doi.org/10.1038/s41586-021-03788-6

Supplementary information

Fair algorithms for selecting citizens' assemblies

In the format provided by the
authors and unedited

Contents

1 Illustration of Definitions with Examples 1

2 Model 3

3 Stratified Sampling 3

4 Desiderata for Sortition in the Political Science Literature 4
- 4.1 Properties of Idealized Sortition . . . 5
- 4.2 Beyond Idealized Sortition, and the Objective of Maximal Fairness . . . 8

5 Related Work on Panel Selection 8

6 Computational Hardness 10

7 Small Optimal Portfolios Exist 11

8 Algorithmic Framework 12
- 8.1 Algorithmic Framework Overview and Context . . . 12
- 8.2 Applications of Framework to Other Problems . . . 13
- 8.3 Conditions for Applying the Framework . . . 14
- 8.4 Definition of Framework . . . 16
- 8.5 Termination and Correctness of Framework . . . 17

9 Fairness Measures 19
- 9.1 Maximizing Egalitarian Welfare . . . 20
- 9.2 Minimizing the Gini Coefficient . . . 21
- 9.3 Minimizing the Atkinson Indices for $0 < \epsilon < 1$ . . . 22
- 9.4 Maximizing Nash Social Welfare . . . 23

10 Description of LexiMin 25
- 10.1 Overview . . . 25
- 10.2 Definition of LexiMin . . . 25
- 10.3 Proofs . . . 27

11 Description of Legacy 29

12 Description of Other Existing Algorithms 29

13 Instances where Legacy is Unfair 30

14 Comparing Legacy and LexiMin on Intersectional Representation 33
- 14.1 Level of Intersectional Representation in Legacy versus LexiMin . . . 34
- 14.2 Explanation for Intersectional Representation in Legacy and LexiMin . 34
- 14.3 Achieving Proportional Representation for Intersections with Our Framework 35

15 Axiomatic Analysis **36**
15.1 Population Monotonicity 36
15.2 Committee Monotonicity 37
15.3 Equal Treatment of Equals 37
15.4 Proportionality 38

1 Illustration of Definitions with Examples

Here, we introduce the definitions and concepts used in this paper through an example *instance*, which is composed of a pool, information about quotas, and a panel size k.

Example instance. Suppose we want to select a panel of size $k = 3$. Let the *features* on which we want to impose *quotas* be female, male, young, and old; and let the lower and upper quotas for each feature be as specified below:

	female	male	young	old
lower quota	1	1	2	1
upper quota	2	2	2	1

Finally, suppose that the *pool* of the instance contains $n = 5$ pool members, which are given with their features:

name	features
Alice	young, female
Bob	old, male
Ciara	young, female
Dan	young, male
Ella	old, female

Panels for the example instance. A *panel* for this instance is any set of 3 pool members in which 1 or 2 are female, 1 or 2 are male, exactly 1 is old, and exactly 2 are young. Therefore, the complete set of panels in this instance is:

$$\mathscr{P} = \{\{\text{Alice, Bob, Ciara}\}, \{\text{Alice, Bob, Dan}\}, \{\text{Ciara, Bob, Dan}\},$$
$$\{\text{Alice, Dan, Ella}\}, \{\text{Ciara, Dan, Ella}\}\}$$

Selection algorithms on this instance. In general, a *selection algorithm* takes in an arbitrary instance and must (randomly) return a panel for that instance. Thus, when a selection algorithm receives our example instance as its input, it must produce one of the panels in $\mathscr{P}$. Now, we compare the behavior of two selection algorithms, LEGACY and LEXIMIN, on this instance. (These algorithms are formally defined in SI 10 and 11, but no knowledge of the algorithms is necessary to follow this example.)

LEGACY* and LEXIMIN each have a different *output distribution* on our instance, both of which are displayed on the left-hand side of the two tables below. While both algorithms return the same set of panels, they differ in how likely each panel is to be

*For one specific way of breaking ties between features (male > female > old > young), which is left unspecified by the algorithm (see SI 11).

selected; for example, LEGACY selects the panel {Alice, Bob, Ciara} with probability 1/6 whereas LEXIMIN selects that panel with probability 1/3.

Each algorithm's output distribution determines the *selection probability* of each pool member. For example, the probability that LEGACY selects a panel containing Ella can be calculated by summing up the output probabilities of both panels that include her: Since LEGACY selects {Alice, Dan, Ella} and {Ciara, Dan, Ella} each with probability 1/6, Ella's selection probability is 1/3. We refer to agents' collective selection probabilities as a *probability allocation*. The probability allocations of the two algorithms are given on the right-hand side of the two tables below.

Fairness measures evaluate the fairness of different probability allocations, which allows us to evaluate whether LEGACY or LEXIMIN is fairer on our instance. One important fairness measure ("egalitarian social welfare"; see SI 9) measures the fairness of a probability allocation by its minimum selection probability. Using this fairness measure, the fairness of LEGACY's probability allocation is 1/3 whereas the fairness of LEXIMIN's probability allocation is 1/2. Since the latter value is higher, the fairness measure judges LEXIMIN to be fairer on the example instance than LEGACY.

In this paper, we develop maximally fair selection algorithms. As it turns out, LEXIMIN is one such algorithm for the fairness measure above, in the sense that, for all instances, and for all other selection algorithms, the minimum selection probability of LEXIMIN will be at least as large as the minimum selection probability of the other algorithm.

LEGACY

Output Distribution	*Probability Allocation*
$\mathbb{P}[\{\text{Alice, Bob, Ciara}\} \text{ selected}] = \frac{1}{6}$	Alice: $\frac{1}{6} + \frac{1}{4} + \frac{1}{6} = \frac{7}{12}$
$\mathbb{P}[\{\text{Alice, Bob, Dan}\} \text{ selected}] = \frac{1}{4}$	Bob: $\frac{1}{6} + \frac{1}{4} + \frac{1}{4} = \frac{2}{3}$
$\mathbb{P}[\{\text{Ciara, Bob, Dan}\} \text{ selected}] = \frac{1}{4}$	Ciara: $\frac{1}{6} + \frac{1}{4} + \frac{1}{6} = \frac{7}{12}$
$\mathbb{P}[\{\text{Alice, Dan, Ella}\} \text{ selected}] = \frac{1}{6}$	Dan: $\frac{1}{4} + \frac{1}{4} + \frac{1}{6} + \frac{1}{6} = \frac{1}{2}$
$\mathbb{P}[\{\text{Ciara, Dan, Ella}\} \text{ selected}] = \frac{1}{6}$	Ella: $\frac{1}{6} + \frac{1}{6} = \frac{1}{3}$

LEXIMIN

Output Distribution	*Probability Allocation*
$\mathbb{P}[\{\text{Alice, Bob, Ciara}\} \text{ selected}] = \frac{1}{3}$	Alice: $\frac{1}{3} + \frac{1}{12} + \frac{1}{4} = \frac{2}{3}$
$\mathbb{P}[\{\text{Alice, Bob, Dan}\} \text{ selected}] = \frac{1}{12}$	Bob: $\frac{1}{3} + \frac{1}{12} + \frac{1}{12} = \frac{1}{2}$
$\mathbb{P}[\{\text{Ciara, Bob, Dan}\} \text{ selected}] = \frac{1}{12}$	Ciara: $\frac{1}{3} + \frac{1}{12} + \frac{1}{4} = \frac{2}{3}$
$\mathbb{P}[\{\text{Alice, Dan, Ella}\} \text{ selected}] = \frac{1}{4}$	Dan: $\frac{1}{12} + \frac{1}{12} + \frac{1}{4} + \frac{1}{4} = \frac{2}{3}$
$\mathbb{P}[\{\text{Ciara, Dan, Ella}\} \text{ selected}] = \frac{1}{4}$	Ella: $\frac{1}{4} + \frac{1}{4} = \frac{1}{2}$

2 Model

An *instance* consists of a set of agents $N = \{1, \ldots, n\}$, a desired panel size k, and a finite set of *features*. Examples of such features could be "female" or "older than 65". Let N_f be the set of agents with feature f. Each feature f is furthermore associated with a lower quota ℓ_f and an upper quota u_f, which specify lower and upper limits on the number of panel seats to be filled by agents in N_f. In a given instance, a *panel* P is any subset of N such that the following integer linear program (ILP) is satisfied by the set of 0–1 indicators x_i that specify whether agent i is in panel P:

$$\begin{aligned}
&\sum_{i \in N} x_i = k && && (P \text{ contains } k \text{ agents}) \\
&\ell_f \leq \sum_{i \in N_f} x_i \leq u_f && \forall \text{ features } f && (P \text{ satisfies all lower and upper quotas}) \\
&x_i \in \{0, 1\} && \forall i \in N && (\text{the } x_i \text{ are binary indicators}).
\end{aligned}$$

In the context of our column-generation framework, we call a set of panels within the same instance a *portfolio*.

To avoid issues of well-definedness, we formally restrict our definition of an instance to include only those in which there exists at least one panel. (In practice, this restriction is unproblematic, since the existence of a panel can be confirmed by checking the satisfiability of the ILP above with an ILP solver before applying a selection algorithm.)

A *selection algorithm* receives an instance as its input and must randomly choose a panel to return. We call the distribution describing the probability with which each panel is returned the selection algorithm's *output distribution* for this instance. If, for a given selection algorithm and input instance, we let the random variable P denote the panel returned by the selection algorithm (its distribution then being the output distribution), the *selection probability* p_i of an agent i is defined as $\mathbb{P}[i \in P]$, and a *probability allocation* is a function mapping each agent $i \in N$ to their selection probability p_i.

Finally, a *fairness measure* for a specific instance is a function $F : [0,1]^n \to (\mathbb{R} \cup \{-\infty\})$ mapping the probability allocations of that instance to a score, where larger scores denote preferable levels of fairness. To avoid artificially reducing the generality of our results, this definition of a fairness measure is specific to one instance. Where we speak of "fairness measures" in the body of the paper and in SI 9 (e.g., "Nash welfare" or "Gini coefficient"), we are formally referring to families of fairness measures, where each family contains one fairness measure for each possible instance.

3 Stratified Sampling

One procedure for selecting random panels that is often discussed is *stratified sampling*. A stratified-sampling procedure is defined by what we will call a *stratification*: a partition of the population into disjoint subgroups (e.g., women, men, people of nonbinary gender), where each subgroup is associated with the number of panel seats they will receive (say, 19,

19, and 2 seats). Then, from each stratum, the procedure uniformly samples the specified number of panel members. Stratified sampling and our selection algorithms similarly strive to ensure descriptive representation. However, our algorithms accept a more flexible range of quotas for expressing constraints on descriptive representation, making them more widely applicable than stratified sampling. For instance, the quota constraints imposed in all ten citizens' assemblies analyzed in this paper cannot be expressed as stratifications.

To understand why the quotas imposed in practice are more general than those imposed by stratified sampling, we first note that the constraints expressed by a stratification can directly be expressed as a system of quotas. This is done by turning each stratum into a feature, and then setting both the feature's lower and upper quota to the desired number of panel seats. By contrast, not every system of quotas can be expressed as a stratification. This is for two reasons: first, whereas practitioners often permit a bit of tolerance between a feature's upper and lower quota, stratified sampling requires specifying the *exact number* of people to be chosen from each stratum. Second, and more fundamentally, quotas are often imposed on overlapping groups (e.g., the groups women and young people, where individuals can belong to both groups at once), whereas all strata must be disjoint.

To see why this restriction limits the generality of stratified sampling, consider an example in which we have overlapping categories gender and age, and want to impose quotas on women, men, people of non-binary gender, young people, and old people. In stratified sampling, one would define six disjoint strata: young women, young men, young people of nonbinary gender, old women, old men, and old people of nonbinary gender. One would then have to specify some exact number of people from each stratum; by contrast, the constraints expressed by quotas on the feature can be much more flexible since they, for example, do not directly constrain the age composition within the group of women.

As illustrated in the above example, one can implement quotas in practical settings by defining the strata to be all intersectional groups. However, this strategy does not extend practicably to the number of feature categories on which quotas are imposed in practice (in our instances, between 4 and 8). This is because imposing quotas on many orthogonal features (e.g, gender, age, region, and education level) would require setting aside a number of seats for exponentially many combinations of these features (e.g., "female, 18–25 years old, London, no diploma"), which would quickly exceed the number of panel seats.

4 Desiderata for Sortition in the Political Science Literature

In this paper, we approach the problem of panel selection from a pragmatic angle. We ask: taking as given the overall panel selection process (sending out invitations uniformly at random, and then using quotas to enforce representativeness), what is the best selection algorithm for practitioners to use?

To identify desirable properties of a selection algorithm, it is natural to take inspiration from political theory, where advantages and disadvantages of sortition have been discussed

in detail[16,23,25,42,47]. However, one should not expect the political theory literature to give concrete instructions for a practical selection algorithm, since the literature focuses on an idealized sortition process that ignores the complications of the real-world settings in which panels must be selected. In particular, the literature assumes that panels can be selected by sampling directly from the population, whereby each member of the population is selected with equal probability and will agree to participate if invited[2,21,24]. We refer to this procedure as *idealized sortition*. Usually, in practice, a large majority of people decline to participate when invited[42].

Though this literature does not immediately prescribe a practical selection algorithm, it informs our approach by identifying the values that should be pursued when designing selection algorithms. In this section, we outline several prominently advocated properties of idealized sortition, discuss how they are or are not conducive to algorithmic implementation, and describe how these properties complement or contradict one another. Ultimately, our approach of making selection probabilities as equal as possible strives for *promotion of equality*, while guaranteeing the achievement of *representativeness* as implemented by practitioners via quotas.

4.1 Properties of Idealized Sortition

Following a model developed by Engelstad[25] and elaborated upon by others[2,24], sortition should simultaneously (1) *promote equality*, (2) *ensure representativeness*, (3) *maximize efficiency*, and (4) *protect against conflict and domination.*

Equality

According to Engelstad, "The strongest normative argument in favour of sortition is linked to the idea of social equality and individual welfare", which stems from the fact that every constituent has an equal selection probability.[25] Subsequent work in political theory has reaffirmed the importance of equal selection probabilities, even if different authors deduce this importance from slightly different ideals: Some[16,21–23] see the equal selection probabilities of idealized sortition as an embodiment of *democratic equality*, the ideal that a democratic decision-making process should give equal consideration to all of its constituents' preferences. Other authors[2,21] stress equal probabilities as the hallmark of (prospect-regarding[48]) *equality of opportunity*. A related argument is made by Stone[23,24]. Rather than seeing equality as the goal in its own right, he views random allocation with equal probability as the only way to satisfy *allocative justice* in the distribution of public offices among constituents who all have equal claims to authority.

As we discuss in the introduction, perfect equality of selection probabilities is not attainable within the constraints of practical sortition. In this paper, we handle this impossibility by proposing a more gradual version of this goal: Subject to achieving descriptive representation, one should make selection probabilities as equal as possible. The view of political office as a good, and of sortition as a means to allocative justice[23], is a natural foundation for the approach of treating panel selection as a problem of fair division (see SI 9).

Representativeness

Another important benefit of ideal sortition is that, with high probability, the composition of the panel will resemble the population along all dimensions of interest[24]. Descriptive representation is a crucial assumption in Fishkin's argument that the result of a deliberative minipublic can reveal the likely outcome of the whole population deliberating[16,22]. In addition to its contribution to the quality of deliberation, descriptive representation is particularly valuable in contexts of mistrust and marginalization[49].

As stated above, the statistical properties of idealized sortition imply that *any possible* division of the population is likely to be represented close to proportionally on the panel, provided that the panel size is sufficiently large. By contrast, no such guarantee can be provided in the realistic setting where constituents decline to participate, which forces practitioners to select specific features for which they want to enforce descriptive representation using quotas. Whereas our approach focuses on making selection probabilities close to equal, we do not sacrifice descriptive representation for this goal. Rather, organizing bodies can still set quotas to ensure a desired level of descriptive representation, and our methods only use the remaining freedom within these constraints to promote equality. In this way, our method allows an assembly organizer to trade off representation and equality by tightening or loosening the quotas.

Efficiency

In comparison to selecting representatives by election, some authors argue that sortition is more efficient because it requires fewer resources[2,25]. For instance, campaigning and organizing elections are not necessary. Arguably, this argument is more specific to the benchmark of elections than to sortition, and subsequent works have put little emphasis on this point[24].

When considering the design of the selection algorithm, the only major resource one might seek to use efficiently is time — namely, the time the algorithm takes to run. Given that the selection of the panel from the pool is only a minor task in organizing and convening a citizens' assembly, as organizers spend much more time recruiting the pool and organizing the deliberation. For this reason, reducing the running time of the algorithm seems a frivolous efficiency. As we show in Table 1, our algorithm LexiMin runs in seconds for most instances and an hour at most. This is significantly longer than the running time of the benchmark algorithm Legacy, but much faster than the process of executing other selection algorithms using dice and spreadsheets, as practiced by some organizations. We take this as an indicator that hours versus minutes of running time is not a significant consideration in terms of efficiency.

Existing algorithms often confront practitioners with a hard trade-off between representation and computational efficiency, since more numerous and tighter quotas may drastically increase the running time of these algorithms. While such a concern cannot be theoretically ruled out for any known algorithm (SI 6), our algorithms delegate the task of finding panels to a state-of-the-art ILP solver, a mature technology routinely used to solve much harder tasks[50] than all panel-selection subtasks we have encountered. Therefore,

we expect our algorithm to allow for much more complex quotas without substantial increases in running time; the fundamental trade-offs between representativeness and equality, of course, persist. Our algorithms also have an advantage in the (undesirable) situation where no panel formed from the pool can satisfy the quotas. Whereas existing algorithms enter an infinite loop in this situation until the user gives up, our algorithms' first call to the ILP solver will immediately reveal that the quotas are infeasible; in these situations, our implementation solves a second ILP to suggest a minimal relaxation of the quotas that can be satisfied.

Protection against Conflict and Domination

A final family of arguments stresses that, if the members of a panel are chosen via idealized sortition, this procedure prevents interested parties from swaying the selection for their benefit[2,25,51]. Stone summarizes these arguments as follows:

> "First, [sortition] can prevent wrongful action on the part of the agent who must select officials. [. . .] Second, it can prevent wrongful action on the part of the officials selected. If the method of selection is in any way predictable, outside interests might bribe or threaten officials into conformity with their wishes. If the method is unpredictable, then such wishes cannot be expressed at least until the results of the lottery become known. [. . .] Finally, competing elites unable to stack the political process in their favor have less to fight about."[24]

In the practical setting of sortition, the additional stages of the selection process (as compared to idealized sortition) inherently create opportunities for dishonest agents to influence the composition and the decisions of the panel in ways that cannot be remedied by a change of selection algorithm. First, with respect to concerns about wrongful action on the part of the officials, the panel organizers wield a lot of influence in sending out the invitations, setting the quotas, and handling the process of selecting the panel from the pool.

More fundamentally, when any selection algorithm enforcing descriptive representation is used, a dishonest pool member can significantly increase their chances of selection by misrepresenting their features. For example, this pool member might pretend to have a different political orientation because they know that people with this orientation are unlikely to participate, and thus are likely to be underrepresented in the pool. Since, on average, the selection algorithm must choose pool members from this group with higher probability, reporting this feature will likely increase the agent's probability of being selected for the panel. So long as practitioners seek to enforce descriptive representation in the presence of unequal rates of participation across subgroups, this type of manipulation seems unavoidable.

If, despite these challenges, one wanted to design a selection algorithm to discourage manipulation, one would have to target a specific kind of manipulation. For instance, for reducing the effect of bribing or intimidating pool members before they are selected,

the algorithm within our framework minimizing the largest selection probabilities might be appropriate. Such an algorithm would increase the cost to the manipulator since any bribed pool member would have a substantial chance of not being selected to the panel, rendering the bribe futile. For other threat models, it would be natural for the selection algorithm to maximize not only the uncertainty of each agent being selected for the panel individually but the uncertainty about the composition of the whole panel. A selection algorithm maximizing this objective of *maximum entropy* could, in principle, be implemented by uniformly drawing sets of k pool members, repeating this process until one set satisfies all quotas. Whether this selection algorithm can be sped up to the degree of being practically relevant is an interesting question for future work.

4.2 Beyond Idealized Sortition, and the Objective of Maximal Fairness

As we have described, a large body of political theory literature characterizes the desiderata and benefits of *idealized* sortition. However, there is also research that engages, as we do in this work, with sortition beyond the idealized assumption that everyone is willing to participate. Such work often mentions *stratified sampling*[2,3,21,42,52] as a sampling method that can be used to reestablish descriptive representation despite differing response rates across subpopulations. For details on stratified sampling and how it relates to our work, see SI 3. In the political theory literature touching on stratified sampling, several authors point out that the benefits of idealized sortition do not perfectly extend to stratified sampling[21,24,42,53]. To our knowledge, however, the literature stops short of proposing more gradual ideals, such as the maximal fairness objective we propose to approximate equality.

5 Related Work on Panel Selection

The algorithmic problem of selecting panels for citizens' assemblies has motivated two previous papers. Both previous papers consider different models of sortition than does this work, and their results are not directly applicable to the practical setting we consider here.

In the first paper, Benadè, Gölz, and Procaccia[27] study a setting closely resembling what we call *idealized sortition* in SI 4 — that is, Benadè et al. assume that the panel-selection procedure can choose any constituent to participate (they assume it has full knowledge of the population) without taking into account that some constituents might not agree to serve on the panel. In this setting, *uniform sampling without replacement* is the most natural selection procedure, and it provides two important benefits: perfect equality of selection probabilities and probabilistic guarantees on the descriptive representation of any arbitrary group in the population. If one wants *deterministic* guarantees on descriptive representation along one specific category of attributes (say, gender), stratified sampling (SI 3) will give such guarantees. Benadè et al. show that such deterministic guarantees can be imposed for certain groups with only marginal deterioration in the representation of other groups. Unfortunately, these results do not extend to the practical setting explored in this paper because, in addition to their unrealistic assumption that all constituents

will participate, the set of quotas that can be imposed via stratified sampling is much more restrictive than those imposed in practice (see SI 3 for details).

The second paper, by Flanigan, Gölz, Gupta, and Procaccia[28], also develops a panel selection procedure, and, unlike Benadè et al., it accounts for the possibility that people invited to the panel may decline to join. Flanigan et al. consider the same general panel-selection pipeline as does this paper, with a uniform sample of the population being invited to participate, invitation recipients self-selecting into a pool of volunteers, and then a selection algorithm choosing the panel from the pool.

The main differences between the paper by Flanigan et al. and ours lies in the level of idealization of the models of sortition, and in the handling of quotas. On both of these counts, this paper engages more directly with the practical setting than does Flanigan et al.: In the present paper, we directly address the problem faced by practitioners when they sample their panel, which means taking as already decided the set of agents who opted into the pool and the quotas imposed by practitioners. As we described in the introduction, with these attributes of the problem already decided, equal selection probabilities are generally not attainable, which is why we focus on achieving equality to the maximum degree possible. By contrast, Flanigan et al. attempt to recover a notion of equal probabilities in an idealized probabilistic model of the panel-selection pipeline. Specifically, in their model, whether an invited agent joins the pool is decided by a biased coin flip, where the success probability of each agent's coin, the agent's *participation probability*, is known to the selection algorithm. Furthermore, quotas are not externally given, but are determined by what the selection algorithm can ensure for the given citizens' assembly. Under these assumptions and further assuming that all participation probabilities lie above a certain minimum bound, Flanigan et al. design a selection algorithm that achieves near-equal *end-to-end probabilities*, i.e., ensures that each agent reaches the panel from the population with similar probability. To do so, it prioritizes selecting those pool members who had the lowest probability of accepting their invitation, essentially canceling out the self-selection bias.

Note that Flanigan et al. and our paper pursue different notions of equality: Their paper aims to equalize the probability of each agent going *from population to panel* (calculated across all possible pools), whereas our paper aims for equality between the selection probabilities of members of a *single* pool. While their notion of equality is conceptually appealing, it is well-defined only relative to their modeling assumption that people decide to join the pool randomly. If one nevertheless wanted to apply their selection algorithm in practice, the agents' "participation probabilities" would have to be estimated using machine learning. Since, depending on these estimates, the selection algorithm might select an individual with much higher or lower selection probability, determining this number based on inherently imprecise techniques raises concerns about algorithmic bias and transparency. Finally, while their selection algorithm ensures some quotas, these guarantees only hold in the limit of very large pools and, even then, the gap between upper and lower quotas remains much looser than the gap between upper and lower quotas typically imposed by practitioners.

6 Computational Hardness

Here we show that, under standard complexity assumptions, there does not exist a selection algorithm (even an unfair one) that runs in polynomial time. At its core, this impossibility is a consequence of the following hardness result:

Theorem 1. *For a given set of agents, panel size, and set of features with associated quotas, it is* NP*-hard to decide whether there exists a panel.*

Proof. By reduction from the NP-complete problem Exact Cover by 3-Sets (X3C)[54]. Fix an X3C instance consisting of a ground set X with $|X| = 3\,q$ and of a collection C of 3-element subsets of X. From this instance, construct an instance of the panel-selection problem as follows: Identify the pool members N with the 3-sets C, create one feature f_x per $x \in X$, and set the panel size k to q. For every feature f_x, we impose quotas $\ell_{f_x} = u_{f_x} = 1$, and we set N_{f_x} to the set of agents whose corresponding 3-set contains x.

It remains to show that there exists a panel iff there exists an exact cover for the X3C instance:
$\Rightarrow$: Suppose that there is a quota-compliant panel $P \subseteq N$. By the definition of the quotas, all features f_x apply to exactly one agent in P. Thus, all elements $x \in X$ occur in exactly one of the three-sets corresponding to P, which means that this collection of 3-sets is an exact cover.
$\Leftarrow$: Let $C' \subseteq C$ be an exact cover for the X3C instance. Note that $|C'| = q = k$ because every set in C' has exactly 3 elements and must cover a universe of size $|X| = 3\,q$. Set the panel P to C'. Since C' covers every element $x \in X$ exactly once, each feature f_x applies to a single agent in P. This shows that the quotas are satisfied. □

Formally, the hardness of this decision problem does not *immediately* contradict the existence of polynomial-time selection algorithms, since our definition of a selection algorithm only allows for *instances* in the input of the algorithm, and instances are required to have at least one panel (SI 2). Nonetheless, the non-existence of polynomial-time algorithms follows as a simple corollary: if a selection algorithm produced a panel in polynomial time *with probability 1*, this would imply $\mathsf{P} = \mathsf{NP}$ (Corollary 1 below), and, even if a selection algorithm succeeded at producing a panel in polynomial time only with constant probability, this would imply $\mathsf{NP} = \mathsf{RP}$ (Corollary 2 below). The latter consequence would in turn imply $\mathsf{NP} = \mathsf{RP} \subseteq \mathsf{P/poly}$ [55] and thus that the polynomial-time hierarchy collapses[56], both of which are widely assumed to be false.

Since polynomial-time selection algorithms are unlikely to exist, this paper studies algorithms that are efficient in practice but whose worst-case running time might scale exponentially.

Corollary 1. *Unless* $\mathsf{P} = \mathsf{NP}$*, there is no selection algorithm that finds a panel in polynomial time (with probability 1).*

Proof. By contrapositive. Suppose that there was a selection algorithm that would return a panel within n^c computation steps for some constant c. Since our definition of instances assumes that all instances possess panels, this hypothetical algorithm may

behave arbitrarily when provided with an input for which no panel exists. Still, this selection algorithm would allow to decide the NP-hard problem from Theorem 1 in polynomial time: Given a set of agents, a panel size, and a set of features, simply simulate the selection algorithm for n^c steps and check whether a quota-compliant panel was returned. Since this polynomial-time algorithm decides an NP-hard problem, the existence of a polynomial-time selection algorithm would imply $\mathsf{P} = \mathsf{NP}$. □

Corollary 2. *Unless* $\mathsf{RP} = \mathsf{NP}$*, there is no selection algorithm that, with constant probability, finds a panel in polynomial time.*

Proof. By contrapositive. Suppose that there was a selection algorithm that, for each instance, would succeed at returning a panel in n^c computation steps (for some constant c) with constant probability. By again simulating this selection algorithm for n^c steps and checking whether a quota-compliant panel was returned, one defines an RP-acceptor for the NP-hard language defined in Theorem 1, implying $\mathsf{RP} = \mathsf{NP}$. □

7 Small Optimal Portfolios Exist

Proposition 1. *Fix an arbitrary instance and a fairness measure F for this instance. If there exists any maximally fair distribution over panels for F, there exists a maximally fair output distribution whose support includes at most $n + 1$ panels.*

Proof. Consider the hypercube $[0, 1]^n$, and associate each dimension with one agent. A panel P can be embedded into this space by its characteristic vector $\vec{v}_P \in \{0, 1\}^n$, whose ith component is one exactly if $i \in P$.

Fix a maximally fair panel distribution, let $\mathscr{P}$ denote its support, and let $\{\lambda_P\}_{P \in \mathscr{P}}$ denote its probability mass function. Note that

$$\vec{p} := \sum_{P \in \mathscr{P}} \lambda_P \, \vec{v}_P$$

is a probability allocation maximizing F, and that it is a convex combination of the $\{\vec{v}_P\}_{P \in \mathscr{P}}$. By Carathéodory's theorem, there is a subset $\mathscr{P}' \subseteq \mathscr{P}$ of size at most $n + 1$ such that $\vec{p}$ still lies in the convex hull of this smaller set. Thus, there are nonnegative real numbers $\{\lambda'_P\}_{P \in \mathscr{P}'}$ adding up to one such that

$$\vec{p} = \sum_{P \in \mathscr{P}'} \lambda'_P \, \vec{v}_P.$$

These λ'_P form the probability mass function of a distribution over at most $n + 1$ panels, which has the same probability allocation $\vec{p}$ as the original maximally fair distribution, which implies that the new distribution is also maximally fair for F. □

8 Algorithmic Framework

In this section, we first summarize the high-level design of our algorithmic framework, how it is situated among existing algorithms and techniques, and how the framework applies to settings other than sortition. We then introduce the notion of a distribution-optimizer family, which encapsulates the information that the framework needs to optimize a fairness measure, and we formally describe the steps of the framework. Finally, we prove the correctness of the framework.

8.1 Algorithmic Framework Overview and Context

At the highest level, each algorithm in our framework maximizes a concave function (the fairness measure). The approach our algorithms take to optimizing these concave functions generalizes a form of *column generation*, an algorithmic technique that is commonly used for solving linear programs with many variables and few constraints.[57] The existing column generation approach for solving such linear programs proceeds as follows: We first consider a version of the linear program in which all but a portfolio consisting of some K of the variables are assumed to be non-basic and set to zero. This restricted version of the program then has only K variables (and the same few constraints as in the original program), so its optimal primal and dual variables can be found efficiently. This primal solution (with zeros for the remaining variables) is then checked for optimality in the entire original program. This is done by looking for a column with negative reduced cost, i.e., a primal variable not currently in the portfolio such that slightly increasing its value from the current value of zero would lead to an increase in the objective. If such a column exists, it is then added to our portfolio of possibly basic variables, and the process is repeated for this slightly larger linear program. Once no such column exists, the solution for the restricted program is already optimal for the entire program.

Our column-generation algorithm applies the same general approach to convex programs satisfying strong duality. We are not aware of many previous papers applying column generation to convex optimization, and the papers we know of use column generation to refine linear approximations of convex functions, rather than directly optimizing the convex function over restricted sets of variables[57,58]. One reason that column generation has not been applied to convex programs themselves might be that general convex programs may not have optimal solutions with few nonzero variables, and thus, column generation might not be faster than direct optimization of the full convex program. As we discuss below, however, the optimization problems considered in this paper have a special structure that ensures the existence of optimal solutions with few nonzero variables, which makes column generation a promising approach.

The convex program we solve, stated in its most general form, is as follows: Let N be a finite set of *entities* (in our case: pool members), and let $\widehat{\mathscr{P}}$ be an implicitly defined (i.e., not explicitly given) family of subsets of N (in our case: quota-compliant panels). Then, we consider a convex program of the following shape:

$$\begin{aligned} &\text{maximize } h(\vec{p}, \vec{x}) && \\ &\text{subject to } g_r(\vec{p}, \vec{x}) \leq 0 && \forall 1 \leq r \leq m \\ &\qquad\qquad \vec{p} \in \mathsf{PossibleMarginals}(\widehat{\mathscr{P}}) && . \end{aligned} \tag{1}$$

Without the constraint in the last row, this would just be a general convex program, with a concave objective function h, m many constraints defined by convex functions g_r, an arbitrary vector of variables $\vec{x}$, and a vector of special variables $\vec{p}$, one per entity. What makes this convex program special is the constraint "$\vec{p} \in \mathsf{PossibleMarginals}(\widehat{\mathscr{P}})$", which expresses that there exists some probability distribution over $\widehat{\mathscr{P}}$ such that the $p_1, \ldots, p_n$ in $\vec{p}$ are the entities' *marginals* induced by that distribution (where an entity's marginal is the probability that a set containing them is drawn from that distribution over $\widehat{\mathscr{P}}$). This last constraint could be easily expanded into additional linear constraints and exponentially many auxiliary variables λ_P, one for the probability mass of each set P in $\widehat{\mathscr{P}}$, but this would require enumerating exponentially many sets in $\widehat{\mathscr{P}}$ and drastically increasing the size of the convex program. As we show in SI 7, Carathéodory's theorem implies that an optimal solution of this expanded program (if one exists) can set all but $|N| + 1$ of the λ_P variables to zero.

Thus, our framework applies column generation to these λ_P variables, repeatedly solving the expanded convex program under the restriction that all λ_P except those in a small portfolio are non-basic and set to zero. Given some additional assumptions (guaranteeing that these restricted programs are solvable and satisfy strong duality), we can define the reduced cost of a set P in $\widehat{\mathscr{P}}$ as a sum of Karush-Kuhn-Tucker (KKT) multipliers corresponding to the set's elements. Thus, our framework reduces optimizing the convex program with the special constraint "$\vec{p} \in \mathsf{PossibleMarginals}(\widehat{\mathscr{P}})$" to the problem of optimizing a linear objective over $\widehat{\mathscr{P}}$ (for finding the column with minimum reduced cost in each iteration of the column generation). When, as in this paper, $\widehat{\mathscr{P}}$ is implicitly defined by an ILP, the framework directly defines an algorithm by using an ILP solver for these subtasks.

8.2 Applications of Framework to Other Problems

Solving convex programs of the form (1) identified above has immediate applications outside of sortition and to combinatorial structures other than quota-compliant panels: For example, Kurokawa, Procaccia, and Shah[39] study the problem of assigning classrooms to charter schools, where the implicit sets in $\widehat{\mathscr{P}}$ correspond to sets of schools that can simultaneously be matched in a bipartite matching with knapsack constraints. While Kurokawa et al. give an algorithm optimizing the leximin criterion in this domain, our framework immediately allows to optimize other fairness measures such as Nash welfare.

A second application lies in kidney exchange, where Roth, Sönmez and Ünver[40] again propose an algorithm for finding the leximin-optimal distribution over matchings, where each edge in the matching connects two donor–patient pairs matched for a 2-way exchange of kidneys. Not only does our framework allow the optimization of fairness measures

other than leximin, but it also extends to the more complex forms of kidney exchange encountered in practice, including longer cyclical exchanges and donation chains initiated by altruistic donors. The literature proposes multiple ILP formulations[59,60] that can be used for this purpose.

While both previous examples optimize individual fairness as their objective, our techniques apply to other convex optimization objectives as well. In SI 14.3, we give an example of an objective that optimizes the descriptive representation of groups rather than aiming for equal selection probabilities between individuals.

8.3 Conditions for Applying the Framework

We now specify conditions that allow a convex program to be solved using our framework. Putting the outline in SI 8.1 into the language of panel selection, the column generation repeatedly (i) optimizes the convex program with the added restriction that the output probabilities of all panels not included in the current portfolio of panels $\mathscr{P}$ are set to zero, and then (ii) uses the KKT multipliers and an ILP solver to identify the panel to add to $\mathscr{P}$ that will allow the greatest marginal increase in fairness, until, eventually, the solution found in (i) is optimal for the unrestricted convex program. We will refer to the restricted convex program for a portfolio $\mathscr{P}$ as $C_{\mathscr{P}}$.

For the column generation to work, all programs $C_{\mathscr{P}}$ it optimizes should have an optimal solution and the KKT conditions should be necessary and sufficient. In particular, having an optimal solution implies that the portfolio must be non-empty from the start (since the output probabilities must add up to one, meaning that they cannot all be zero). We formalize these assumptions in a structure called a *distribution-optimizer family*:

Definition 1 (distribution-optimizer family)**.** A *distribution-optimizer family (DOF)* $\mathscr{C}$ for an instance is a family of convex programs that is fully specified by the tuple $(\mathscr{P}_{init}, t, h, \{g_r\}_r)$, where the four elements of this tuple are as follows:

- $\mathscr{P}_{init}$ is a non-empty portfolio of panels of the instance,
- $t \in \mathbb{N}_0$ is the number of auxiliary variables in each convex program,
- $h : ([0,1]^n \times \mathbb{R}^t) \to \mathbb{R}$ is a differentiable concave function (the objective of the convex programs), and
- the $g_r : ([0,1]^n \times \mathbb{R}^t) \to \mathbb{R}$ for $1 \leq r \leq m$ are some number $m \in \mathbb{N}_0$ of affine functions (defining auxiliary constraints in the convex programs).[†]

This tuple defines a family of convex programs $\mathscr{C} = \{C_{\mathscr{P}}\}_{\mathscr{P} \supseteq \mathscr{P}_{init}}$, which includes one program $C_{\mathscr{P}}$ for each portfolio $\mathscr{P}$ in the instance such that $\mathscr{P} \supseteq \mathscr{P}_{init}$. Each such convex program $\mathscr{P}$ has variables $\{\lambda_P\}_{P \in \mathscr{P}}$ (representing the output probabilities of panels P),

[†]The functions g_r can be differentiable convex rather than affine as long as the strong duality of all convex problems $C_{\mathscr{P}}$ below is still ensured, for instance by Slater's condition.

$\vec{p} = \{p_i\}_{i \in N}$ (representing the selection probabilities of agents i), and $\vec{x}$ (a t-dimensional vector of real-valued auxiliary variables), and the convex program is defined as follows:

$$
\begin{aligned}
&\text{maximize } h(\vec{p}, \vec{x}) && \\
&\text{subject to } \sum_{P \in \mathscr{P}} \lambda_P = 1 && && \text{(output probabilities add to 1)} \\
&\qquad p_i = \sum_{\substack{P \in \mathscr{P} \\ i \in P}} \lambda_P && \forall i \in N && \text{(marginals are sums of output probabilities)} \\
&\qquad g_r(\vec{p}, \vec{x}) \le 0 && \forall 1 \le r \le m && \text{(auxiliary constraints)} \\
&\qquad \lambda_P \ge 0 && \forall P \in \mathscr{P} && \text{(output probabilities are nonnegative).}
\end{aligned}
$$

For $\mathscr{C}$ to be a DOF for the instance, in addition to being defined by a tuple as specified above, it must hold that all convex programs $C_{\mathscr{P}}$ for $\mathscr{P} \supseteq \mathscr{P}_{init}$ are solvable (i.e., they are feasible and the optimal value is attained).

The algorithmic framework takes as input a specific instance and a DOF $\mathscr{C}$ for this instance, and the framework then uses column generation to decide which convex programs from $\mathscr{C}$ to run in what order to find the maximally fair distribution. Therefore, to use the framework to optimize a specific fairness measure F on a given instance, one simply needs to find a DOF for that instance that optimizes F (if one exists). The following definition formally connects a fairness measure with a DOF that optimizes it:

Definition 2 (implementation of a fairness measure by a DOF)**.** For a specific instance, a fairness measure F for the instance is *implemented* by a DOF $\mathscr{C} = \{C_{\mathscr{P}}\}_{\mathscr{P} \supseteq \mathscr{P}_{init}}$ if, for any portfolio $\mathscr{P} \supseteq \mathscr{P}_{init}$, each optimal solution to $C_{\mathscr{P}}$ yields the probability mass function $\{\lambda^*_P\}_{P \in \mathscr{P}}$ of a distribution that is maximally fair according to F among all distributions over the support $\mathscr{P}$.

As we show below, for each DOF $\mathscr{C}$ of an instance, it is easy to construct a fairness measure F for that instance that is implemented by the DOF, by setting $F(\vec{p}) \coloneqq \sup\{h(\vec{p}, \vec{x}) \mid \vec{x} \in \mathbb{R}^t, \forall 1 \le r \le m.\ g_r(\vec{p}, \vec{x}) \le 0\}$, with the convention that $\sup \emptyset = -\infty$. However, $\mathscr{C}$ simultaneously implements other fairness measures whose optimization leads to the same optima (for example, the same DOF might implement the product of probabilities and the sum of their logarithms).

Proposition 2. *For a fixed instance, a DOF $\mathscr{C} = \{C_{\mathscr{P}}\}_{\mathscr{P} \supseteq \mathscr{P}_{init}}$ for this instance implements the fairness measure F specified by*

$$F(\vec{p}) \coloneqq \sup\{h(\vec{p}, \vec{x}) \mid \vec{x} \in \mathbb{R}^t, \forall 1 \le r \le m.\ g_r(\vec{p}, \vec{x}) \le 0\}.$$

Proof. Fix an instance and fix a portfolio $\mathscr{P} \supseteq \mathscr{P}_{init}$. Denote the optimal objective value of $C_{\mathscr{P}}$ by obj^*, and note that, by the definition of a DOF, this optimal value is attained.

We must show that, for any optimal solution of $C_{\mathscr{P}}$, the λ^*_P are the probability mass function of a distribution that is maximally fair according to F among distributions over

the support $\mathscr{P}$, i.e., that the $\vec{p}^*$ optimize F. We will show this in two steps: In step (1), we show that, if $\vec{p}$ is the probability allocation corresponding to an optimal solution of $C_{\mathscr{P}}$, then $F(\vec{p}) = obj^*$. In step (2), we show that, for each probability allocation $\vec{p}$ that can be obtained by a distribution over $\mathscr{P}$, it holds that $F(\vec{p}) \leq obj^*$. Together, these steps imply that a probability allocation $\vec{p}$ is optimal according to F (among probability allocations of distributions over $\mathscr{P}$) iff $F(\vec{p}) = obj^*$, and that this is the case for the probability allocation of each panel distribution given by an optimal solution of $C_{\mathscr{P}}$.

Step (1). Consider an optimal solution $\vec{\lambda}^*, \vec{p}^*, \vec{x}^*$ to $C_{\mathscr{P}}$. Note that its objective value must be obj^*. Furthermore, note that if we added constraints fixing each selection probability p_i to p_i^* and each panel probability λ_P to λ_P^* to the convex program $C_{\mathscr{P}}$, the optimal objective value of the restricted problem would still be obj^* and would still be attained. Since $F(\vec{p})$ is defined as the optimal objective value of this restricted problem, $F(\vec{p}) = obj^*$.

Step (2). Now, consider any probability allocation $\vec{p}^*$ that is the result of a distribution $\mathscr{D}$ over $\mathscr{P}$. By fixing $\vec{p}$ in $C_{\mathscr{P}}$ to $\vec{p}^*$ and by fixing $\vec{\lambda}$ to the probability mass function of $\mathscr{D}$, $C_{\mathscr{P}}$ simplifies to the optimization problem defining $F(\vec{p})$, which means that the optimal objective value obj^* of the full convex program $C_{\mathscr{P}}$ is at least $F(\vec{p})$. □

8.4 Definition of Framework

As described above, the algorithmic framework is an algorithm that takes as input an instance and a DOF of that instance. The framework then computes a distribution over panels that is maximally fair with respect to the fairness measure implemented by the DOF, and then samples this distribution to select the final panel. The full algorithm is specified below:

Algorithm 1: FRAMEWORK

Input: an instance and a corresponding DOF $\mathscr{C} = \{C_{\mathscr{P}}\}_{\mathscr{P} \supseteq \mathscr{P}_{init}}$
Output: a randomly chosen panel for the instance

$\mathscr{P} \leftarrow \mathscr{P}_{init}$;
while *true* **do**
  let $\vec{\lambda}^*, \vec{p}^*, \vec{x}^*$ denote an optimal solution for $C_{\mathscr{P}}$, and let μ_r^* be the dual value for each constraint $g_r(\vec{p}, \vec{x}) \leq 0$ at this optimum;
  for $i \in N$ **do**
    $\eta_i^* \leftarrow \frac{\partial}{\partial p_i} h(\vec{p}^*, \vec{x}^*) - \sum_{r=1}^{m} \mu_r^* \frac{\partial}{\partial p_i} g_r(\vec{p}^*, \vec{x}^*)$;
  $P_{new} \leftarrow$ panel P maximizing $\sum_{i \in P} \eta_i^*$, found by ILP (P need not be in $\mathscr{P}$);
  $P_{old} \leftarrow$ some panel $P \in \mathscr{P}$ such that $\lambda_P^* > 0$;
  if $\sum_{i \in P_{old}} \eta_i^* \geq \sum_{i \in P_{new}} \eta_i^*$ **then**
    $\mathscr{D} \leftarrow$ distribution over $\mathscr{P}$ with probability mass function $\vec{\lambda}^*$;
    return panel drawn from $\mathscr{D}$;
  else
    $\mathscr{P} \leftarrow \mathscr{P} \cup \{P_{new}\}$;

8.5 Termination and Correctness of Framework

It remains to show that the above algorithm always terminates (Theorem 2) and that it selects panels in a maximally fair way (Theorem 3). In the proofs of these theorems, we will extensively use the Karush-Kuhn-Tucker (KKT) conditions for the convex optimization problems $C_{\mathscr{P}}$. Consider a specific instance and a specific DOF $\mathscr{C} = \{C_{\mathscr{P}}\}_{\mathscr{P} \supseteq \mathscr{P}_{init}}$ for this instance. Then, we denote

- the dual variable of the constraint $\sum_{P \in \mathscr{P}} \lambda_P = 1$ by η_0,
- the dual variables of the constraints $p_i = \sum_{P \in \mathscr{P} : i \in P} \lambda_P$ by η_i,
- the dual variables of the constraints $g_r(\vec{p}, \vec{x}) \leq 0$ by μ_r, and
- the dual variables of the constraints $\lambda_P \geq 0$ by ν_P.

Since $C_{\mathscr{P}}$ satisfies strong duality, the following KKT conditions are necessary and sufficient for optimality:

$$\sum_{P \in \mathscr{P}} \lambda_P = 1 \tag{2}$$

$$p_i = \sum_{\substack{P \in \mathscr{P} \\ i \in P}} \lambda_P \qquad \forall i \in N \tag{3}$$

$$g_r(\vec{p}, \vec{x}) \leq 0 \qquad \forall 1 \leq r \leq m \tag{4}$$

$$\lambda_P \geq 0 \qquad \forall P \in \mathscr{P} \tag{5}$$

$$\mu_r \geq 0 \qquad \forall 1 \leq r \leq m \tag{6}$$

$$\nu_P \geq 0 \qquad \forall P \in \mathscr{P} \tag{7}$$

$$\mu_r \, g_r(\vec{p}, \vec{x}) = 0 \qquad \forall 1 \leq r \leq m \tag{8}$$

$$\nu_P \, \lambda_P = 0 \qquad \forall P \in \mathscr{P} \tag{9}$$

$$\left(\sum_{i \in P} \eta_i \right) + \nu_P = \eta_0 \qquad \forall P \in \mathscr{P} \tag{10}$$

$$\eta_i = \frac{\partial}{\partial p_i} h(\vec{p}, \vec{x}) - \sum_{r=1}^{m} \mu_r \, \frac{\partial}{\partial p_i} g_r(\vec{p}, \vec{x}) \qquad \forall i \in N \tag{11}$$

$$\nabla_{\vec{x}} \, h(\vec{p}, \vec{x}) = \sum_{r=1}^{m} \mu_r \, \nabla_{\vec{x}} \, g_r(\vec{p}, \vec{x}) \tag{12}$$

In the following proofs, we will denote the set of all panels of the instance by $\widehat{\mathscr{P}}$.

Theorem 2. *Algorithm 1 terminates.*

Proof. Fix the input instance and the DOF $\mathscr{C} = \{C_{\mathscr{P}}\}_{\mathscr{P} \supseteq \mathscr{P}_{init}}$. It suffices to show that $\mathscr{P}$ grows in every iteration since it is always a subset of the finite set $\widehat{\mathscr{P}}$ of all panels of the instance. More specifically, we need to show that, whenever the if branch in Line 8 is not taken, P_{new} was not yet in $\mathscr{P}$.

Note that, in Line 5 of Algorithm 1, the η_i^* are set equal the dual variables η_i at the optimum of $C_{\mathscr{P}}$ by Eq. (11).[‡] From complementary slackness (9) and the precondition $\lambda_{P_{old}} > 0$ (Line 7), we know that $\nu_{P_{old}} = 0$, and thus, by Eq. (10), that

$$\sum_{i \in P_{old}} \eta_i^* = \eta_0^* = \left(\sum_{i \in P'} \eta_i^* \right) + \nu_{P'}^* \geq \sum_{i \in P'} \eta_i^*$$

for all $P' \in \mathscr{P}$, where the last step uses Eq. (7). Since, by assumption, the if branch in Line 8 was not taken, we know that $\sum_{i \in P_{new}} \eta_i^* > \sum_{i \in P_{old}} \eta_i^* \geq \sum_{i \in P'} \eta_i^*$ for all $P' \in \mathscr{P}$, which shows that P_{new} was not yet in $\mathscr{P}$. □

Theorem 3. *Fix any instance, and let a DOF $\mathscr{C} = \{C_{\mathscr{P}}\}_{\mathscr{P} \supseteq \mathscr{P}_{init}}$ for this instance implement a fairness measure F. Then, when Algorithm 1 is called with the instance and $\mathscr{C}$, its output distribution is maximally fair according to F.*

Proof. Consider the point in the execution of Algorithm 1 just before returning, when the algorithm defines the distribution $\mathscr{D}$ in Line 9. Since all computation steps so far are deterministic, and since the algorithm subsequently just returns a panel drawn from $\mathscr{D}$, $\mathscr{D}$ is the output distribution of the algorithm when given these inputs. It remains to show that $\mathscr{D}$ is maximally fair according to F.

Since $C_{\mathscr{P}}$ (for the value of $\mathscr{P}$ when the algorithm is in Line 9) satisfies strong duality, we know that the variables $\vec{\lambda}^*, \vec{p}^*, \vec{x}^*, \vec{\mu}^*, \vec{\eta}^*$ can be extended by variables $(\nu_P^*)_{P \in \mathscr{P}}$ and η_0^* to satisfy the KKT conditions of $C_{\mathscr{P}}$.

We will extend these variables for $C_{\mathscr{P}}$ to variables satisfying the KKT conditions for the larger convex program $C_{\widehat{\mathscr{P}}}$. In this extension, we preserve the values of all variables already present from $C_{\mathscr{P}}$, and set $\lambda_P^* := 0$ and $\nu_P^* := \eta_0^* - \sum_{i \in P} \eta_i^*$ for all $P \in \widehat{\mathscr{P}} \setminus \mathscr{P}$.

Next, we show that this assignment satisfies the KKT conditions for $C_{\widehat{\mathscr{P}}}$. Most of the conditions directly follow from the assumption that the KKT conditions hold for $C_{\mathscr{P}}$ because all variables in the equation remained the same (Eqs. (4), (6), (8), (11) and (12); and Eqs. (5), (7), (9) and (10) for all $P \in \mathscr{P}$). The first two conditions (Eqs. (2) and (3)) are preserved because all newly introduced λ_P^* are zero. Clearly, all λ_P^* are nonnegative (Eq. (5)). Similarly, the added ν_P^* for $P \in \widehat{\mathscr{P}} \setminus \mathscr{P}$ are nonnegative (Eq. (7)) because the algorithm took the if branch in Line 8, which means that

$$\sum_{i \in P} \eta_i^* \leq \sum_{i \in P_{new}} \eta_i^* \leq \sum_{i \in P_{old}} \eta_i^* \leq \left(\sum_{i \in P_{old}} \eta_i^* \right) + \nu_{P_{old}}^* = \eta_0^*.$$

Complementary slackness (Eq. (9)) is satisfied because the added λ_P^* are zero, and condition (10) holds by the definition of the new ν_P^*. This shows that all KKT conditions for $C_{\widehat{\mathscr{P}}}$ are satisfied, implying the constructed assignment is optimal.

[‡] Thus, the algorithm could alternatively have been written as taking the η_i^* directly as the optimal dual variable values of the η_i. We do not do so to avoid ambiguity in the sign of η_i^* and to stress that $\sum_{i \in P} \eta_i^*$ can be understood as a reduced cost of the column λ_P, based on the gradient of the convex function.

Since $\mathscr{C}$ implements the fairness measure F, the distribution whose probability mass function is given by the constructed λ_P^* is maximally fair among distributions over the support $\widehat{\mathscr{P}}$, and therefore maximally fair among all output distributions. Since, in extending the assignment, we only added λ_P^* variables with value 0, $\mathscr{D}$ is equal to this maximally fair distribution. □

9 Fairness Measures

In different sub-areas of fair division, researchers have developed metrics measuring how fairly utility is distributed over individuals by a given allocation of a resource[30,37]. By casting the problem of panel selection as a fair-division problem below, we demonstrate how these metrics can be used to quantify the fairness of probability allocations produced by selection algorithms:

> *Consider each quota-compliant panel in a given instance to be a distinct public good, and suppose that society can select exactly one of these goods, possibly through a random lottery. Each agent in the pool has value 1 for any panel on which they are featured, and value 0 for any panel on which they are not featured; and an agent's utility for a lottery over panels is their expected value for the drawn panel.*

In this setup, each pool member's utility is exactly their selection probability, which is determined by the selected lottery over panels. Therefore, metrics for measuring the fairness of a utility profile in the fair division literature can be applied to measure the fairness of a distribution over panels by giving them a probability allocation as their input rather than a vector of utilities.

Now, we describe multiple metrics from the fair-division literature that can be used as fairness measures in the panel-selection setting. In the subsections below, we show how each of these fairness measures can be maximized using our framework.

Egalitarian social welfare[35]: Maximize the lowest selection probability, $\min_{i \in N} p_i$.

Gini coefficient[36,37]: Minimize half of the relative mean absolute difference,

$$\frac{\sum_{i\in N}\sum_{j\in N}|p_i - p_j|}{2\,n\,\sum_{i\in N} p_i}.$$

Atkinson indices[37,38]: For a given parameter $\epsilon \in (0,1)$, minimize

$$1 - \frac{n}{\sum_{i\in N} p_i}\left(\frac{\sum_{i\in N} p_i^{1-\epsilon}}{n}\right)^{1/(1-\epsilon)}.$$ [§]

[§]Note that, in our setting, minimizing the Atkinson index for $\epsilon = 1$ coincides with maximizing Nash welfare.

Nash social welfare[30]**:** Maximize the product of selection probabilities, $\prod_{i \in N} p_i$.

Recall that our definition of a fairness measure (SI 2) assumes that higher values indicate higher levels of fairness. Thus, the sign of the Gini coefficient and the Atkinson indices needs to be inverted to obtain a fairness measure according to our formal definition.

Given that Nash social welfare and egalitarian social welfare are listed as fairness measures above, one might expect utilitarian social welfare (i.e., the sum of selection probabilities) to also appear. However, since the sum of selection probabilities is equal to k for all probability allocations, utilitarian welfare is a constant function in our setting, which can hardly be considered a measurement of fairness.

Another important formalization of fairness from the fair-division literature is the *leximin criterion*[30], which we implement in our algorithm LexiMin. Recall that the leximin objective not only maximizes the lowest selection probability (as does egalitarian welfare), but then breaks ties in favor of the second-lowest selection probability, the third-lowest selection probability and so on. Since this objective cannot be represented as the maximization of a single real-valued score[30], leximin cannot formally be expressed as a fairness measure according to our definition (SI 2). Nevertheless, the leximin criterion defines a weak ordering of probability allocations, which is enough to define a maximally fair probability allocation. Specifically, to compare two probability allocations $\{p_i\}_{i \in N}$ and $\{q_i\}_{i \in N}$, one represents each by a vector of probability values sorted in non-decreasing order and compares these vectors using the lexicographic order.

9.1 Maximizing Egalitarian Welfare

For any instance, the egalitarian-welfare fairness measure is defined by

$$F_{egal}(\vec{p}) = \min_{i \in N} p_i.$$

Let P_o be an arbitrary panel for the instance, which can be found by ILP. We will show that the DOF $\mathscr{C}_{egal} = \{C_{\mathscr{P}}\}_{\mathscr{P}}$ defined by the tuple

$$\langle \{P_o\}, 1, (\vec{p}, x) \mapsto x, \{(\vec{p}, x) \mapsto x - p_i\}_{i \in N} \rangle$$

implements F_{egal}. Since t, h, and the g_r can be read from the convex optimization problem, it is more convenient to implicitly specify them via the parametric convex program $C_{\mathscr{P}}$:

$$\begin{aligned}
\text{maximize } & x \\
\text{such that } & \sum_{P \in \mathscr{P}} \lambda_P = 1 \\
& p_i = \sum_{\substack{P \in \mathscr{P} \\ i \in P}} \lambda_P && \forall i \in N \\
& x - p_i \leq 0 && \forall i \in N \\
& \lambda_P \geq 0 && \forall P \in \mathscr{P}.
\end{aligned}$$

Proposition 3. *For each instance, $\mathscr{C}_{egal}$ is a DOF.*

Proof. We must show that, for each $\mathscr{P} \supseteq \mathscr{P}_{init} = \{P_o\}$, the optimal value of $C_{\mathscr{P}}$ is attained. Since $C_{\mathscr{P}}$ is a linear program, this reduces to showing that the program is feasible and bounded.

For any $\mathscr{P} \supseteq \{P_o\}$, $C_{\mathscr{P}}$ is feasible by setting $\lambda_{P_o} \coloneqq 1$, $\lambda_P \coloneqq 0$ for all other $P \in \mathscr{P}$, by setting the p_i according to their functional dependency on the λ_P, and by setting $x \coloneqq 0$. Furthermore, the optimal value is bounded from above since, in any valid assignment, fixing an arbitrary agent $i \in N$,

$$x \le p_i = \sum_{\substack{P \in \mathscr{P} \\ i \in P}} \lambda_P \le \sum_{P \in \mathscr{P}} \lambda_P = 1. \qquad \square$$

Proposition 4. *For each instance, the fairness measure F_{egal} for this instance is implemented by the DOF $\mathscr{C}_{egal}$ for this instance.*

Proof. By Proposition 2, $\mathscr{C}_{egal}$ implements the fairness measure F given by

$$\begin{aligned} F(\vec{p}) &= \sup\{x \mid x \in \mathbb{R}, \forall i \in N.\ x - p_i \le 0\} \\ &= \sup\{x \mid x \in \mathbb{R}, \forall i \in N.\ x \le p_i\} \\ &= min_{i \in N} p_i. \end{aligned} \qquad \square$$

9.2 Minimizing the Gini Coefficient

For any instance, the Gini-coefficient fairness measure is defined by

$$F_{gini}(\vec{p}) = -\frac{\sum_{i \in N} \sum_{j \in N} |p_i - p_j|}{2\, n \sum_{i \in N} p_i}.$$

Again, let P_o be an arbitrary panel of the instance, found by ILP. We will show that the DOF $\mathscr{C}_{gini} = \{C_{\mathscr{P}}\}_{\mathscr{P} \supseteq \mathscr{P}_{init}}$ implements F_{gini}, where $\mathscr{C}_{gini}$ is defined by setting $\mathscr{P}_{init} \coloneqq \{P_o\}$ and by implicitly defining t, h, and the g_r through the following convex program $C_{\mathscr{P}}$:

$$\begin{aligned} \text{maximize} \quad & -\sum_{i<j\in N} x_{i,j} & \\ \text{such that} \quad & \sum_{P \in \mathscr{P}} \lambda_P = 1 & \\ & p_i = \sum_{\substack{P \in \mathscr{P} \\ i \in P}} \lambda_P & \forall i \in N \\ & -x_{i,j} + p_i - p_j \le 0 & \forall i < j \in N \\ & -x_{i,j} - p_i + p_j \le 0 & \forall i < j \in N \\ & \lambda_P \ge 0 & \forall P \in \mathscr{P}, \end{aligned}$$

where "$i < j \in N$" is short-hand for requiring that $i, j \in N$ and that i precedes j in a canonical ordering over agents.

Proposition 5. *For each instance, $\mathscr{C}_{gini}$ is a DOF.*

Proof. We must show that, for each $\mathscr{P} \supseteq \mathscr{P}_{init} = \{P_o\}$, the optimal value of $C_{\mathscr{P}}$ is attained. Since $C_{\mathscr{P}}$ is a linear program, it suffices to show that the program is feasible and bounded.

For any $\mathscr{P} \supseteq \{P_o\}$, $C_{\mathscr{P}}$ is feasible by setting $\lambda_{P_o} := 1$, $\lambda_P := 0$ for all other $P \in \mathscr{P}$, by setting the p_i according to their functional dependency on the λ_P, and by setting all $x_{i,j}$ to 1 (since then, e.g., $-x_{i,j} + p_i - p_j \leq -1 + p_i \leq 0$). Furthermore, the optimal value is bounded from above since, in any valid assignment, the $x_{i,j}$ are constrained to be at least $p_i - p_j$ and at least $-p_i + p_j = -(p_i - p_j)$, which means that all $x_{i,j}$ are nonnegative and, thus, that $-\sum_{i<j\in N} x_{i,j}$ cannot be positive. □

Proposition 6. *For each instance, the fairness measure F_{gini} for this instance is implemented by the DOF $\mathscr{C}_{gini}$ for this instance.*

Proof. By Proposition 2, $\mathscr{C}_{gini}$ implements the fairness measure F given by

$$\begin{aligned}
F(\vec{p}) &= \sup\left\{ -\textstyle\sum_{i<j\in N} x_{i,j} \;\middle|\; \begin{array}{l} \{x_{i,j}\}_{i<j\in N} \in \mathbb{R}^{\binom{n}{2}}, \\ \forall i,j \in N.\; x_{i,j} \geq p_i - p_j \text{ and } x_{i,j} \geq p_j - p_i \end{array} \right\} \\
&= \sup\left\{ -\textstyle\sum_{i<j\in N} x_{i,j} \;\middle|\; \begin{array}{l} \{x_{i,j}\}_{i<j\in N} \in \mathbb{R}^{\binom{n}{2}}, \\ \forall i,j \in N.\; x_{i,j} \geq |p_i - p_j| \end{array} \right\} \\
&= -\textstyle\sum_{i<j\in N} |p_i - p_j| \\
&= -\frac{\sum_{i\in N}\sum_{j\in N} |p_i - p_j|}{2} \\
&= F_{gini}(\vec{p})\, n \textstyle\sum_{i\in N} p_i \\
&= F_{gini}(\vec{p})\, n\, k.
\end{aligned}$$

Thus, $\mathscr{C}_{gini}$ implements a fairness measure that is just F_{gini} times the positive constant $n\,k$. Since multiplying a fairness measure by a positive constant does not change which probability allocations maximize the fairness measure, $\mathscr{C}_{gini}$ also implements F_{gini}. □

9.3 Minimizing the Atkinson Indices for $0 < \epsilon < 1$

For a fixed instance, and a fixed constant $\epsilon \in (0, 1)$, the Atkinson-index fairness measure is defined by

$$F_{atkinson}(\vec{p}) = \frac{n}{\sum_{i\in N} p_i} \left(\frac{\sum_{i\in N} p_i^{1-\epsilon}}{n} \right)^{1/(1-\epsilon)} - 1.$$

Again, let P_o be an arbitrary panel of the instance, found by ILP. We will show that the DOF $\mathscr{C}_{atkinson} = \{C_{\mathscr{P}}\}_{\mathscr{P}\supseteq\mathscr{P}_{init}}$ implements $F_{atkinson}$, where $\mathscr{C}_{atkinson}$ is defined by setting $\mathscr{P}_{init} := \{P_o\}$ and by implicitly defining t, h, and the g_r through the following convex program $C_{\mathscr{P}}$:

$$
\begin{aligned}
\text{maximize } & \sum_{i \in N} p_i^{1-\epsilon} \\
\text{such that } & \sum_{P \in \mathscr{P}} \lambda_P = 1 \\
& p_i = \sum_{\substack{P \in \mathscr{P} \\ i \in P}} \lambda_P && \forall i \in N \\
& \lambda_P \geq 0 && \forall P \in \mathscr{P}.
\end{aligned}
$$

Proposition 7. *For each instance, $\mathscr{C}_{gini}$ is a DOF.*

Proof. We must show that, for each $\mathscr{P} \supseteq \mathscr{P}_{init} = \{P_o\}$, the optimal value of $C_{\mathscr{P}}$ is attained. Since there are no auxiliary constraints, feasibility is trivial given that $\mathscr{P}$ is nonempty. Since there are no auxiliary variables, all variables are naturally bounded in $[0, 1]$. Since the domain of valid assignments for $\vec{\lambda}$ and $\vec{p}$ is bounded and closed, thus compact, the continuous function h attains its maximum on this domain. □

Proposition 8. *For each instance, the fairness measure $F_{atkinson}$ for this instance is implemented by the DOF $\mathscr{C}_{atkinson}$ for this instance.*

Proof. By Proposition 2, $\mathscr{C}_{atkinson}$ implements the fairness measure F given by

$$
\begin{aligned}
F(\vec{p}) &= \sup\{\textstyle\sum_{i \in N} p_i^{1-\epsilon}\} \\
&= \textstyle\sum_{i \in N} p_i^{1-\epsilon} \\
&= n \, \left(k/n \, (F_{atkinson}(\vec{p}) + 1)\right)^{1-\epsilon}.
\end{aligned}
$$

Since F can be obtained by composing $F_{atkinson}$ with a strictly monotone function, it has the same maximally fair probability allocations. This shows that $\mathscr{C}_{atkinson}$ also implements $F_{atkinson}$. □

9.4 Maximizing Nash Social Welfare

For a fixed instance, and a fixed constant $\epsilon \in (0, 1)$, the Nash-welfare fairness measure is defined by

$$F_{nash}(\vec{p}) = \prod_{i \in N} p_i.$$

Using an ILP solver, one can determine all agents $i \in N$ who appear on any panel. If any agent i does not appear on a panel, their selection probability must be 0, which means that F_{nash} is constant on all probability allocations and can be maximized by deterministically returning any panel.[¶] Thus, without loss of generality, we assume that each agent $i \in N$ is contained in a panel P_i, which can be found by n ILP calls.

[¶]In practice, one would instead remove all agents from the pool who are not contained in any panel, and optimize Nash social welfare for the resulting instance with fewer agents.

Consider the family of concave programs $\mathscr{C}_{nash} = \{C_{\mathscr{P}}\}_{\mathscr{P} \supseteq \mathscr{P}_{init}}$ where $\mathscr{P}_{init} = \{P_i \mid i \in N\}$ and the convex program $C_{\mathscr{P}}$ is given as

$$\begin{aligned}
\text{maximize} \quad & \sum_{i \in N} \log p_i \\
\text{such that} \quad & \sum_{P \in \mathscr{P}} \lambda_P = 1 \\
& p_i = \sum_{\substack{P \in \mathscr{P} \\ i \in P}} \lambda_P && \forall i \in N \\
& \lambda_P \geq 0 && \forall P \in \mathscr{P}.
\end{aligned}$$

We will show that, by inserting this family of concave programs into our framework, the framework optimizes F_{nash}. A formal complication is that the objective function h defined above is not real-valued for all probability allocations, since it is $-\infty$ whenever one selection probability is zero. Thus, this family does not *quite* fit into our definition of a DOF. However, the proof of optimality of the framework still goes through given that the C_P can be optimized by a convex-program solver and that the optimal values of all $C_{\mathscr{P}}$ are real-valued:

Proposition 9. *For each $C_{\mathscr{P}}$ for some $\mathscr{P} \supseteq \mathscr{P}_{init}$, the optimal objective value is real-valued and attained.*

Proof. Fix some $\mathscr{P} \supseteq \mathscr{P}_{init}$. We will first show that the optimal objective value is not $-\infty$. Indeed, consider the distribution obtained by selecting each panel P_i with probability $1/|\mathscr{P}_{init}|$. Since, by construction, each agent is contained in at least one panel in $\mathscr{P}_{init}$, each selection probability p_i is at least $1/|\mathscr{P}_{init}| \geq 1/n$. This means that an objective value of $n \log(1/n) > -\infty$ can be attained and that the constraints are feasible. Furthermore, it shows that any probability allocation that selects some agent i with probability strictly less than $1/n^n$ cannot be optimal, because its objective value $\sum_{j \in N} \log p_j \leq \log p_i < n \log(1/n)$ is lower than the previous value.

It remains to show that the optimal objective value can be attained. Consider the space of all valid assignments $\vec{\lambda}, \vec{p}$, which is bounded and closed. By the argument above, we do not change the optimal objective value of $C_{\mathscr{P}}$ by further restricting the program with the constraints $p_i \geq 1/n^n$ for all i, and the space of assignments for $\vec{\lambda}, \vec{p}$ still stays compact in this operation. Since $h(\vec{p}) = \sum_{i \in N} \log p_i$ is real-valued and continuous on this space, its maximum is attained. □

Proposition 10. *For each instance, plugging $\mathscr{C}_{nash}$ into the framework yields an output distribution that is maximally fair according to F_{nash}.*

Proof. Following the reasoning of the proof of Theorem 3, one shows that the probability mass function of the output distribution is optimal according to $C_{\bar{\mathscr{P}}}$ in $\mathscr{C}_{nash}$. By the reasoning of Proposition 2, this yields a probability allocation that maximizes the fairness measure F given by

$$\begin{aligned} F(\vec{p}) &= \sup\{\textstyle\sum_{i \in N} \log p_i\} \\ &= \textstyle\sum_{i \in N} \log p_i \\ &= \log(F_{nash}(\vec{p})). \end{aligned}$$

Since this is a strictly monotone transformation of F_{nash}, the output distribution must also be maximally fair for F_{nash}. □

10 Description of LexiMin

10.1 Overview

As we discussed in SI 9, leximin is not formally a fairness measure according to our definition, which means that it cannot be optimized with a single application of our framework. Instead, we repeatedly invoke the framework for different auxiliary DOFs as follows: In the first application of the framework, we maximize the minimum probability. Subject to fixing the selection probability of a specific set of agents at this value (we discuss below how these agents are chosen), we then maximize the minimum selection probability among all other agents in a second application of the framework. We continue by fixing the selection probabilities of more and more agents to their value in the leximin allocation until all probabilities are fixed.

The crucial step in the algorithm is knowing which agents' probabilities to fix in each iteration. For example, the first invocation of the framework, which maximizes the minimum selection probability, might result in a probability allocation in which multiple agents have this minimum selection probability. In this case, not all of these agents must have this minimum selection probability in the leximin-optimal distribution, so it is not obvious whose selection probability should be fixed. As in previous work[61], complementary slackness allows us to identify at least one agent in each iteration whose selection probability must be minimal across *all* distributions optimizing the current iteration's DOF. Since all leximin-optimal distributions are optimal for the current DOF, we can fix these agents' selection probabilities.

In the following, we first define the auxiliary DOFs and the LexiMin algorithm. Then, we prove the correctness of the algorithm.

10.2 Definition of LexiMin

To define the algorithm, we must first specify the auxiliary DOFs used by it. Each auxiliary DOF is a family $\mathscr{C}_{aux}(R, \rho, \mathscr{P}_{init})$ parametrized by a set $R \not\subseteq N$ of agents and by a function $\rho : R \to [0, 1]$, which together represent that the selection probability of each agent $i \in R$ has been fixed to $\rho(i)$; and by an initial portfolio.

For a set of agents $R \subsetneq N$, a function $\rho \colon R \to [0, 1]$, and a non-empty portfolio $\mathscr{P}_{init}$, the DOF $\mathscr{C}_{aux}(R, \rho, \mathscr{P}_{init}) = \{C_{\mathscr{P}}\}_{\mathscr{P} \supseteq \mathscr{P}_{init}}$ for an instance is defined via the initial portfolio $\mathscr{P}_{init}$ and the following optimization problem $C_{\mathscr{P}}$:

$$
\begin{aligned}
&\text{maximize } x \\
&\text{such that } \sum_{P \in \mathscr{P}} \lambda_P = 1 \\
&\qquad p_i = \sum_{\substack{P \in \mathscr{P} \\ i \in P}} \lambda_P && \forall i \in N \\
&\qquad x - p_i \leq 0 && \forall i \in N \setminus R \\
&\qquad p_i - \rho(i) \leq 0 && \forall i \in R \\
&\qquad \rho(i) - p_i \leq 0 && \forall i \in R \\
&\qquad \lambda_P \geq 0 && \forall P \in \mathscr{P}.
\end{aligned}
$$

We will show in Lemma 1 below that, whenever LexiMin applies the framework to such a $\mathscr{C}_{aux}(R, \rho, \mathscr{P}_{init})$, it indeed defines a DOF. Furthermore, we show in Lemma 2 that this DOF maximizes $\min_{i \in N \setminus R} p_i$ among all probability allocations that select each $i \in R$ with probability exactly $\rho(i)$.

We now define the LexiMin algorithm:

Algorithm 2: LexiMin

Input: an instance

Output: a randomly chosen panel for the instance

1 $\mathscr{P}_{lexi} \leftarrow \{$arbitrary panel P_o found by ILP$\}$;
2 $R \leftarrow \emptyset$;
3 initialize empty function $\rho : R \rightarrow [0, 1]$;
4 $\mathscr{D} \leftarrow$ deterministic distribution with value P_o (for analysis only);
5 **while** $R \subsetneq N$ **do**
6   execute Algorithm 1 up to Line 9 with the instance and the DOF $\mathscr{C}_{aux}(R, \rho, \mathscr{P}_{lexi})$ as input; set $\vec{p}^*, \vec{\mu}^*, \mathscr{D}$ to their final values inside the subprocedure call; and set $\mathscr{P}_{lexi}$ to the final value of $\mathscr{P}$ in the call;
7   **for** $i \in N \setminus R$ **do**
8   **if** $\mu_r^* > 0$ *for* r *corresponding to constraint* $x - p_i \leq 0$ **then**
9             $R \leftarrow R \cup \{i\}$;
10            $\rho(i) \leftarrow p_i^*$;
11 **return** panel drawn from $\mathscr{D}$;

Note that, since $N \neq \emptyset$, the loop is executed at least once and the initialization of $\mathscr{D}$ in Line 4 will never be used. However, this initialization will be convenient in the proof of correctness. In Theorems 4 and 5, we prove that the selection algorithm terminates and that it is indeed maximally fair according to the leximin criterion.

Our practical implementation of LexiMin deviates from the formal specification of Algorithm 2 by the following modifications, which speed up the practical runtime while preserving optimality: (i) implementing Lines 3 to 5 of Algorithm 1 purely in terms of the dual linear program, by (ii) solving these linear programs using interior-point

barrier methods (which typically allow to fix more probabilities per iteration) and by (iii) initializing $\mathscr{P}_{lexi}$ in Line 1 with multiple panels found through a multiplicative-weight heuristic.

10.3 Proofs

Lemma 1. *Whenever Algorithm 2 applies the framework with an instance and $\mathscr{C}_{aux}(R, \rho, \mathscr{P}_{lexi})$, the latter is a DOF for the instance.*

Proof. Fix any $\mathscr{P} \supseteq \mathscr{P}_{init} = \mathscr{P}_{lexi}$. We must show that the optimal value of $C_{\mathscr{P}}$ is attained. Because $C_{\mathscr{P}}$ is a linear program, it suffices to show that it is feasible and bounded.

Since $R \subsetneq N$, the objective value x is clearly bounded from above since, for any $i \in N \setminus R$,

$$x \leq p_i = \sum_{\substack{P \in \mathscr{P} \\ i \in P}} \lambda_P \leq \sum_{P \in \mathscr{P}} \lambda_P = 1.$$

It remains to show that $C_{\mathscr{P}}$ is feasible. Indeed, in the very first application of the framework, $\mathscr{P}_{init}$ is chosen to contain any arbitrary panel P_o. Since $R = \emptyset$, $\mathscr{C}_{aux}(\emptyset, \rho, \{P_o\})$ is equal to $\mathscr{C}_{egal}$ as defined in SI 9.1 and a DOF by Proposition 3.

In subsequent applications, $\mathscr{P}_{init}$ is chosen to be the portfolio $\mathscr{P}_{lexi}$ produced by the previous iteration. In this case, R and ρ were updated such that the final values $\vec{\lambda}^*$ and $\vec{p}^*$ of the previous application of the framework are a feasible solution to the optimization problem of the current application (setting λ_P of all $P \notin \mathscr{P}_{init}$ to zero). □

Lemma 2. *Whenever Algorithm 2 applies the framework with an instance and the DOF $\mathscr{C}_{aux}(R, \rho, \mathscr{P}_{lexi})$, the DOF implements the fairness measure F given by*

$$F(\vec{p}) = \begin{cases} \min_{i \in N \setminus R} p_i & \text{if } \forall i \in R.\ p_i = \rho(i) \\ -\infty & \text{otherwise.} \end{cases}$$

Proof. By Proposition 2, the DOF implements the fairness measure F' given by

$$F'(\vec{p}) = \sup\{x \mid x \in \mathbb{R}, \forall i \in N \setminus R.\ p_i \geq x, \forall i \in R.\ p_i = \rho(i)\}.$$

We will show that $F' = F$, by fixing some $\vec{p}$ and showing that $F'(\vec{p}) = F(\vec{p})$. If $\forall i \in R.\ p_i = \rho(i)$, then

$$F'(\vec{p}) = \sup\{x \mid x \in \mathbb{R}, \forall i \in N \setminus R.\ p_i \geq x\} = \min_{i \in N \setminus R} p_i.$$

Else, i.e., if $p_i \neq \rho(i)$ for some $i \in R$, then $F'(\vec{p}) = \sup \emptyset = -\infty$. □

Theorem 4. *Algorithm 2 terminates.*

Proof. It is enough to show that the size of $R \subseteq N$ grows in each iteration of the while loop.

Recall that the KKT stationarity condition on $\vec{x}$ (12) states that

$$\nabla_{\vec{x}} h(\vec{p}, \vec{x}) = \sum_{r=1}^{m} \mu_r \, \nabla_{\vec{x}} g_r(\vec{p}, \vec{x}).$$

Note that $\frac{\partial}{\partial x}(x - p_i) = 1$, that $\frac{\partial}{\partial x}(p_i - \rho(i)) = \frac{\partial}{\partial x}(\rho(i) - p_i) = 0$, and that $\frac{\partial}{\partial x} h(\vec{p}, x) = \frac{\partial}{\partial x} x = 1$. Thus, the stationarity condition simplifies to

$$1 = \sum_{r \text{ constraint of shape } x - p_i \leq 0} \mu_r.$$

This shows that at least one of the optimal dual variables μ_r^* for a constraint $x \leq p_i$ must be positive, and that the size of R increases in Line 9. □

Theorem 5. *For any instance, the output distribution of Algorithm 2 on this instance is maximally fair according to the leximin criterion.*

Proof. We will prove the following invariant for the while loop in Line 5 of Algorithm 2: (1) for all agents $i \in R$, $\rho(i)$ is this agent's selection probability in the leximin-optimal probability allocation,[‖] and (2) $\mathscr{D}$ is a distribution over $\mathscr{P}_{lexi}$ giving each $i \in R$ selection probability exactly $\rho(i)$.

Before proving the loop invariant, we show that it implies the correctness of the algorithm. Indeed, when the while loop exits, $R = N$, which means that ρ specifies the whole leximin-probability allocation by part (1) of the invariant. By part (2) of the invariant, the distribution $\mathscr{D}$, which is the output distribution of the algorithm, implements the best possible probability allocation according to the leximin criterion and is therefore itself maximally fair.

It is easy to see that the loop invariant holds when we enter the loop for the first time since it is nearly vacuous for $R = \emptyset$. It remains to show that each iteration of the loop preserves the loop invariant.

It follows from the definition of the leximin criterion and part (1) of the invariant that the leximin-optimal probability allocation maximizes $x = \min_{i \in N \setminus R} p_i$ among all possible probability allocations guaranteeing $p_i = \rho(i)$ for all $i \in R$. By Lemma 2 and Theorem 3, the output distribution of Algorithm 1 with the arguments as provided in Line 6 also is a solution to this maximization problem. Fix $p_i^*, \mu_r^*, \mathscr{D}$, and $\mathscr{P}_{lexi}$ as in Line 6, and call the optimal objective value $x^* = \min_{i \in N \setminus R} p_i^*$.

To re-establish part (1) of the invariant, we must look at the agents $i \in N \setminus R$ whose selection probability gets fixed to p_i^* in Line 10. Note that the dual variable μ_r^* is positive, and, as shown in the proof of Theorem 3, that this is also an optimal assignment for the dual variable in the problem $C_{\overline{\mathscr{P}}}$ in $\mathscr{C}_{aux}(R, \rho, \mathscr{P}_{lexi})$, ranging over all panels. By complementary slackness (8), the positivity of μ_r^* implies that the constraint $x \leq p_i$ is

‖ The leximin-optimal probability allocation is uniquely determined as shown for example in Theorem 3.7 by Kurokawa et al.[39].

tight, meaning that $\rho(i)$ is set to $p_i = x^*$. While it follows from the application of our framework that *some* agent in $N \setminus R$ must have probability x^* in the leximin-optimal probability allocation, it is not immediately clear that this must be the case for the specific agent i. However, $\mu_r^* > 0$ furthermore implies that the constraint $x \leq p_i$ is tight in *all* optimal solutions to $C_{\bar{\mathscr{P}}}$ (see p. 95 of Schrijver[62]), and all the leximin-optimal distributions are such optimal solutions. This shows that agent i's selection probability is fixed to the probability x^* the agent receives in the leximin-optimal probability allocation, as claimed. Part (2) of the loop invariant follows from the fact that the distribution returned by the call to Algorithm 1 satisfies all fixed probabilities and has support $\mathscr{P}_{lexi}$. □

11 Description of Legacy

The Legacy algorithm proceeds in k rounds, adding one pool member to the panel per round. Each round begins by calculating the *need* of each feature f remaining in the pool, which is defined as

$$need_f := \frac{\ell_f - (\#\text{ panel members already selected with feature } f)}{\#\text{ remaining pool members with feature } f}.$$

Note that $need_f$ may be negative. After calculating $need_f$ for all features, the algorithm chooses a feature f_{max} with maximal need and draws the next panel member uniformly from the remaining pool members with feature f_{max}. The selected panel member is then removed from the pool.

After adding this person to the panel, the panel might, for one or more features f, now contain u_f many people with feature f. In this case, all remaining pool members with feature f are removed from the pool. If this procedure produces a quota-compliant panel after the kth round, this panel is returned. Else, i.e., if the pool becomes empty in an earlier round or if the final panel violates some quotas, the algorithm is restarted from the beginning.

For intuition, note that the panel resulting from this procedure can violate quotas for several different reasons: it could happen that the kth person is selected but not all the lower quotas are satisfied yet, or the algorithm could run out of people of a certain type before fulfilling a lower quota if some of these agents were previously removed when an upper quota was reached.

The selection algorithms developed by other practitioner organizations generally follow the same structure of selecting panel members one by one, determining which agents to choose next based on myopic heuristics. We describe these algorithms in the following section.

12 Description of Other Existing Algorithms

All existing algorithms we have heard about are listed below, and all select panel members one-by-one, backtracking or restarting if they encounter a quota violation. In most cases, a fully specified algorithmic description was not available, but we did obtain a high-level

sketch of how each of these algorithms selects the next panel member. We list these algorithms by organization below, and describe their basic functionality:

G1000: G1000's algorithm works similarly to LEGACY, except that it calculates the need of a feature as a difference rather than as a ratio.

IFOK: IFOK's algorithm is also generally similar to LEGACY, but, rather than choosing only the next panel member from the feature with greatest need and then recalculating need, the entire lower quota of the feature with highest need is filled at once.

Nexus: The algorithm used by Nexus focuses less on features but rather selects uniformly from the pool, removing people from the pool once any of their features has reached its upper quota.

MASS LBP: MASS LBP typically uses tight lower and upper quotas on all their features. Their algorithm uses one bin for each feature category (e.g., gender, ethnicity, ...), each initially filled with k balls labeled with the correct distribution of features of this category (e.g., $k/2$ women and $k/2$ men). In every round, one ball is drawn from each bin. If a member of the pool has exactly this set of features, the pool member is chosen as the next panel member. Since this will often not be possible, MASS LBP employs elaborate (and not fully formalized) procedures of redrawing balls and backtracking on earlier picks.[4]

13 Instances where LEGACY is Unfair

In this section, we define a family of instances on which LEGACY selects one individual much more rarely than the others, even though it would be possible to select all agents with equal probability. For illustration, we present one specific instance before defining the family:

Say that we want to select an assembly of $k = 200$ people that includes at least 99 of each category: women, men, liberals, and conservatives. Let the pool consist of 1,000 conservative men, 999 liberal women, and 1 conservative woman. Note that the algorithm that selects 100 uniformly drawn women and 100 uniformly drawn men satisfies the quotas and selects each pool member with equal probability 10%. By contrast, one can verify that the LEGACY algorithm alternates between seeing liberals and men as the categories with highest need, skipping the conservative woman in each of the first 198 draws. Depending on how ties are broken for the last two panel selections (when all lower quotas are met), the conservative woman might even be chosen with probability 0, but with at most probability 0.2%.

Definition 3 below generalizes this example to a wide range of agent numbers and panel sizes. In all these instances, it is possible to select all agents with equal probability k/n. At the same time, depending on tie breaking, LEGACY might select the conservative woman with probability probability as low as zero (Proposition 12) or up to a selection probability in $\mathscr{O}(1/n)$ (Proposition 13). Note that the ratio of this latter probability and

the probability of equal selection k/n can be made arbitrarily small by scaling up the size of the instance (Corollary 3).

Definition 3. Let n and k be even, positive integers, such that $n \geq 2\,k$. Define the instance *Alternate*(n, k) as follows:

- Set the panel size to k.
- Let there be four features: female (f), male (m), liberal (ℓ), and conservative (c). Let each feature have a lower quota of $k/2 - 1$ and an upper quota of k (i.e., there are effectively no upper quotas).
- Let the pool consist of $n/2$ conservative men, $n/2 - 1$ liberal women, and one conservative woman.

Proposition 11. *For any instance Alternate(n, k), it is possible to select each agent with equal probability k/n.*

Proof. Consider the selection algorithm that chooses $k/2$ women and $k/2$ men, each uniformly at random without replacement. It is easy to verify that this procedure will select each woman and each man with probability $\frac{k/2}{n/2} = k/n$. Moreover, this procedure will always select exactly $k/2$ women, exactly $k/2$ men, between $k/2$ and $k/2+1$ conservatives and between $k/2 - 1$ and $k/2$ liberals; which means that all panels produced by the procedure satisfy the quotas. □

Lemma 3. *When* LEGACY *is called on Alternate(n, k),*

- *all picks numbered* $1, 3, 5, \ldots, k - 3$ *are liberal women, and*
- *all picks numbered* $2, 4, 6, \ldots, k - 2$ *are conservative men.*

Proof. By strong induction on the number $i = 0, 1, \ldots, k - 3$ of panel members picked so far.

Suppose that i is even. We will show that the next pick (the $i+1$th) is a liberal woman. By the induction hypothesis, $s_\ell = i/2$ liberal women and $s_m = i/2$ conservative men have been selected so far. The need for each of the four features is

$$\begin{aligned} need_f &= (k/2 - 1 - s_\ell)/(n/2 - s_\ell) \\ need_m &= (k/2 - 1 - s_m)/(n/2 - s_m) \\ need_\ell &= (k/2 - 1 - s_\ell)/(n/2 - 1 - s_\ell) \\ need_c &= (k/2 - 1 - s_m)/(n/2 + 1 - s_m). \end{aligned}$$

Note that all the numerators are positive and equal, and that all the denominators are positive. Thus, the feature with highest need is the feature with lowest denominator, which is ℓ. Thus, the algorithm selects a liberal, which can only be a woman.

Now, suppose that i is odd. We will show that the next pick (the $i+1$th) is a conservative man. By the induction hypothesis, $s_\ell = \lceil i/2 \rceil$ liberal women and $s_m = \lfloor i/2 \rfloor$ conservative men have been selected so far. The need for each of the four features is

$$\begin{aligned} need_f &= (k/2 - 1 - s_\ell)/(n/2 - s_\ell) \\ need_m &= (k/2 - 1 - s_m)/(n/2 - s_m) \\ need_\ell &= (k/2 - 1 - s_\ell)/(n/2 - 1 - s_\ell) \\ need_c &= (k/2 - 1 - s_m)/(n/2 + 1 - s_m). \end{aligned}$$

It is easy to see that $need_m > need_c$ and that $need_\ell > need_f$. Furthermore,

$$\begin{aligned} \frac{need_m}{need_\ell} &= \frac{(n/2 - 1 - s_\ell)/(n/2 - s_m)}{(k/2 - 1 - s_\ell)/(k/2 - 1 - s_m)} \\ &= \frac{(n/2 - 2 - s_m)/(n/2 - s_m)}{(k/2 - 2 - s_m)/(k/2 - 1 - s_m)} \\ &= \frac{1 - 2/(n/2 - s_m)}{1 - 1/(k/2 - 1 - s_m)} \\ &= \frac{1 - 2/(n/2 - s_m)}{1 - 2/(k - 2 - 2\,s_m)} \\ &\geq \frac{1 - 2/(k - s_m)}{1 - 2/(k - 2 - 2\,s_m)} && (k \leq n/2) \\ &> 1. \end{aligned}$$

This shows that the feature with highest need is male (m), which implies that the next pick must be a conservative man. □

Proposition 12. *If* LEGACY *breaks ties between features with equal need in a worst-case way, the conservative woman in Alternate(n, k) is selected with zero probability.*

Proof. By Lemma 3, the conservative woman is never among the first $k - 2$ picks. For the $k - 1$th pick, all features are exactly at their lower quota and therefore have a need of 0. The implementation breaks ties in the order in which the features are specified, so might break the tie in favor of liberals (ℓ), which would mean that another liberal woman is selected. Then, in the last pick, the categories liberal and female have negative need because they exceed their lower quota, whereas the categories male and conservative still have a need of 0. If the tie is broken in favor of male, the last selection is a conservative man. Since all quotas are satisfied, the algorithm does not restart but returns this panel. Assuming the above tie-breaking decisions, the conservative woman will never be selected. □

Proposition 13. *No matter how* LEGACY *breaks ties between features with equal need, the conservative woman in Alternate(n, k) is selected with probability at most $8/n$.*

Proof. Again, Lemma 3 shows that the conservative woman is never among the first $k - 2$ picks. At the time of $k - 1$th pick, there are $n/2 - (k/2 - 1)$ women left in the pool and

$n/2+1-(k/2-1)$ conservatives. At the time of the kth pick, these numbers are at still least $n/2-k/2$ and $n/2+1-k/2$. Since all quotas are already satisfied by the first $k-2$ picks, the algorithm does not restart. Thus, by a union bound over the last two picks, the selection probability of the conservative woman is at most

$$\frac{1}{n/2-(k/2-1)}+\frac{1}{n/2-k/2}\leq\frac{2}{n/2-k/2}\leq\frac{2}{n/2-n/4}=\frac{8}{n}. \qquad \square$$

Corollary 3. *Even assuming best-case tie breaking between features with equal need, for every $\epsilon>0$, there is an instance where it is possible to select agents with equal probability k/n, but where* Legacy *selects some agent with probability at most $\epsilon\,k/n$.*

Proof. Let k be an even integer larger than $8/\epsilon$, and let $n=2\,k$. By Proposition 11, it is possible to select each agent with equal probability k/n. By Proposition 13, the selection probability of the conservative woman is at most $8/n\leq\epsilon\,k/n$. $\square$

14 Comparing Legacy and LexiMin on Intersectional Representation

While most of the paper is concerned with representation guarantees to *individuals*, in this section, we consider how the selection algorithms Legacy and LexiMin impact the representation of *groups*. Note that both selection algorithms must satisfy quotas, and thus both algorithms will proportionally represent the groups delineated by the features. Therefore, we direct our focus to groups defined by the *intersection* of multiple features (e.g., "young woman", where "young" and "woman" are the features being intersected). Throughout this section, we study each group's *panel share*, which is the expected value of the fraction of the pool filled with that group's members (i.e., the sum of selection probabilities of all of its members divided by k). Ideally, to provide perfectly accurate descriptive representation, each intersectional group's panel share would be equal to its share in the population.

A priori, we would expect neither LexiMin nor Legacy to accurately represent intersectional groups in proportion to their population share, since neither of these algorithms has precise information about the population shares of these groups, and they do not explicitly try to give these groups accurate representation. Instead, the panel share of an intersectional group will likely arise incidentally from the algorithms' efforts to ensure the satisfaction of quotas. The panel shares given by LexiMin may additionally be impacted by its effort to equalize the selection probabilities between pool members, which could result in groups' panel shares being closer to their representation levels in the pool.

In this section, we investigate how accurately each algorithm represents intersectional groups in one real-world instance, *sf(e)*. We find that the algorithms give similar levels of intersectional representation overall, and in fact, the level of representation given to each *specific group* is similar across the two algorithms. We then find evidence suggesting an explanation for this similarity: for both algorithms, it seems that the panel shares of

intersectional groups mainly reflect the quotas, rather than the frequency of groups in the pool. We conclude by suggesting two ways in which our framework can be used for explicitly promoting the accurate representation of intersectional groups.

We perform this analysis on only a single dataset because the analysis requires knowledge of the population shares of all intersectional groups. Effectively, this requires a separate survey dataset, conducted on the exact population underlying the panel and including all features protected by the assembly's quotas. For the instance *sf(e)*, a nation-wide panel in the UK, we make use of the 2016 European Social Survey (ESS)[63].[**] We restrict our analysis to combinations defined by two features ("2-intersections") because, for intersections of three or more features, many intersectional groups are so small that we do not expect the ESS to represent their true population shares.

14.1 Level of Intersectional Representation in LEGACY versus LEXIMIN

ED Figure 4 compares the deviation from proportional representation given to each individual 2-intersection by each respective algorithm. The histograms on the margins of the plot show that these deviations are concentrated around zero, indicating that both algorithms give fairly accurate representation to most intersectional groups. Nonetheless, a few 2-intersections are misrepresented by more than 15 percentage points, i.e., their true and proportional panel shares differ by more than 0.15. We compare the relative performance of LEGACY and LEXIMIN using the *mean squared error*, i.e., the mean (calculated over all 2-intersections) of the squared difference between the population share and the panel share. Smaller mean squared errors indicate more accurate descriptive representation. We find that this error value is essentially the same for both algorithms, indicating that they achieve essentially the same level of representation for these intersectional groups: LEGACY gives a mean squared error of $1.40 \cdot 10^{-3}$, and LEXIMIN one of $1.36 \cdot 10^{-3}$.

14.2 Explanation for Intersectional Representation in LEGACY and LEXIMIN

As the scatter plot in the center of ED Figure 4 shows, the points track closely with a line of slope equal to 1, indicating that not only do LEXIMIN and LEGACY achieve similar overall levels of intersectional representation, but that they over- and underrepresent the same groups by similar amounts. Indeed, the mean squared error between a group's panel share for LEGACY and a group's panel share for LEXIMIN is $1.99 \cdot 10^{-4}$, implying that the panel shares of a given group by the two algorithms are more closely related to each other than to the population share. This suggests that another property associated with the 2-intersections might determine the group's panel share more accurately than the population share, across both selection algorithms.

[**] The ESS data is preprocessed as described in Appendix D.2 of Flanigan et al.[28], and the population shares of intersectional groups computed from this data are included in our code repository.

One property of intersectional groups that might influence their panel shares across both algorithms is their share in the pool. This is particularly relevant — and of potential concern — for LexiMin, whose efforts to equalize individuals' selection probabilities might push it to overrepresent groups that are overrepresented in the pool. Our findings do not substantiate these concerns: as measured by the mean squared error, the panel share given by either algorithm is less closely related to the pool share (Legacy: $2.60 \cdot 10^{-3}$, LexiMin: $2.37 \cdot 10^{-3}$) than to the population share, and, while this distance is smaller for LexiMin than for Legacy, the difference is small.

In contrast to the pool share, we find that a group's panel share as naïvely extrapolated from the quotas *does* closely mirror the panel shares we observe resulting from either algorithm. We extrapolate from the quotas to predicted panel shares by defining the *quota share* (related to the ratio product defined in the methods section "Individuals Rarely Selected by Legacy") of the intersection of features f_1 and f_2 as

$$\frac{\ell_{f_1} + u_{f_1}}{2\,k} \cdot \frac{\ell_{f_2} + u_{f_2}}{2\,k}.$$

This quota share can be understood as a naïve estimation of the population share of the 2-intersection, assuming that features f_1 and f_2 are uncorrelated. We find that the mean squared error between the 2-intersections' panel shares and their quota shares (Legacy: $1.69 \cdot 10^{-4}$, LexiMin: $1.76 \cdot 10^{-4}$) are substantially smaller than the error between panel and population shares, and on the same scale as the distance between the panel shares of both algorithms. These findings suggest that the descriptive representation of an intersectional group is more directly determined by the quotas of its constituent features rather than its share in the population or the pool. These results also suggest that the panel produced by both selection algorithms do not automatically replicate the correlation of features found in the population, but rather tends towards a composition in which features are closer to uncorrelated. If this phenomenon generalizes across citizens' assemblies, this would be an argument in favor of explicitly promoting intersectional representation, as we do in the following subsection.

14.3 Achieving Proportional Representation for Intersections with Our Framework

In the above, we observed that neither selection algorithm happens to represent intersectional groups at a high level of accuracy. This suggests that, if the accurate representation of intersectional groups is an important consideration, one should attempt to incorporate this goal (and the data about population shares) explicitly into the algorithm. Below, we present two ways of using our framework to make the expected representation of intersectional groups closer to proportional:

First, one could enforce hard constraints on the representation of these intersectional groups by imposing lower and upper quotas on them, just as is traditionally done for single-feature groups. In fact, practitioners already do this on occasion for intersectional groups of particular interest. The downside of this approach is that it poorly scales to large numbers of intersections, because it is difficult to estimate how tight these quotas

can be before quota-compliant panels cease to exist. Moreover, the number and tightness of these quotas trade off against the goal of equalizing selection probabilities in ways that can be difficult to predict.

A method that side-steps these downsides is to promote the proportional representation of intersectional groups as a soft constraint, by incorporating it into the fairness measure. Specifically, if one has a collection of groups g, each of which is associated with a set of pool members N_g and a population share $q_g \in [0, 1]$, maximizing the concave expression

$$- \sum_{\text{groups } g} \left(q_g - \sum_{i \in N_g} p_i / k \right)^2$$

minimizes the mean square error between the panel shares given by the algorithm and the population shares. This term can either be turned into a distribution-optimizer family (Definition 1) that minimizes this error without consideration for individual selection probabilities, or it can be added to the objective function of another DOF, and the user can then optimize a linear combination of the chosen fairness measure and this mean squared error term. In defining this objective, the user can choose how strongly they want to prioritize intersectional representation over individual fairness by modifying the coefficients of the linear combination.

15 Axiomatic Analysis

In searching for fair selection algorithms, we found the approach of optimizing quantitative measures of fairness more useful than the axiomatic method. The main reason for this is that a range of standard axioms of fair division are either trivially satisfied by all selection algorithms or impossible to satisfy by any selection algorithm, making them useless for delineating "good" algorithms. For example, no selection algorithm can guarantee *envy freeness*[30] on all instances, since the quotas of most instances preclude selecting every agent with equal probability k/n. *Pareto efficiency*[64], on the other hand, is trivially satisfied by all selection algorithms, since the sum of selection probabilities is always k. In SI 15.1 and 15.2 below, we show that the relational axioms *population monotonicity*[64] and *committee monotonicity*[65] are also impossible to guarantee.

Two classical axioms that *are* meaningful in comparing selection algorithms are *equal treatment of equals*[30] and a form of *proportionality*[66]. In SI 15.3 and 15.4, respectively, we show, via standard arguments, that LexiMin satisfies both of these axioms.

15.1 Population Monotonicity

Definition 4 (population monotonicity)**.** A selection algorithm guarantees *population monotonicity* if, when additional agents are added to an instance, the selection probability of all previously existing agents weakly decreases.

Theorem 6. *No selection algorithm can guarantee population monotonicity.*

Proof. Fix a selection algorithm A, and consider an instance with six agents, $k = 3$, and four features. We indicate an agent's feature membership as a four-element Boolean vector, where the ith entry of the vector indicates whether the agent exhibits feature i. Using this convention, let the agents' features be given as as agent 1: $(1, 0, 0, 0)$, agent 2: $(0, 1, 0, 0)$, agent 3: $(1, 1, 0, 0)$, agent 4: $(0, 0, 1, 0)$, agent 5: $(0, 0, 0, 1)$, and agent 6: $(0, 0, 1, 1)$. For each feature f, set the lower quota ℓ_f to 1 and the upper quota to 3 (i.e., there is effectively no upper quota). This instance has quota-compliant panels, for example the panel $\{\text{agent } 1, \text{agent } 2, \text{agent } 6\}$. Consider the probability allocation of A on this instance. Since $k = 3$, agents 1, 2, 4, and 5 cannot all simultaneously have zero selection probability. W.l.o.g., assume that agent 1 has positive selection probability.

Now, consider a modified instance in which agent 6 is removed. In this instance, one verifies that the only quota-compliant panel is {agent 3, agent 4, agent 5}, which means that A must select agent 1 with zero probability. This violates population monotonicity since adding back agent 6 would strictly increase the selection probability of agent 1. □

15.2 Committee Monotonicity

Definition 5 (committee monotonicity)**.** A selection algorithm guarantees *committee monotonicity* if, when an instance is modified by increasing k (and remains an instance), the selection probability of all agents weakly increase.

Proposition 14. *No selection algorithm can guarantee committee monotonicity.*

Proof. Consider an instance with three agents and two features. Define the features of the agents using the vector notation from the proof of Theorem 6 as agent 1: $(1, 0)$, agent 2: $(0, 1)$, and agent 3: $(1, 1)$. If the lower and upper quotas for both features are set to 1, the only panel for $k = 1$ is $\{\text{agent } 3\}$, and the only panel for $k = 2$ is $\{\text{agent } 1, \text{agent } 2\}$. Thus, any selection algorithm must strictly decrease agent 3's selection probability when going from $k = 1$ to $k = 2$. □

15.3 Equal Treatment of Equals

Definition 6 (equal treatment of equals)**.** A selection algorithm guarantees *equal treatment of equals* if, for every instance and for every pair of agents i_1, i_2 that have exactly the same set of features, i_1 and i_2 are selected with equal probability.

Theorem 7. LexiMin *guarantees equal treatment of equals.*

Proof. Fix an instance and two agents i_1, i_2 with equal features. Let $\mathscr{D}$ denote the output distribution of LexiMin on this instance. For the sake of contradiction, assume that i_1 is selected with a probability p_1 strictly higher than the selection probability p_2 of i_2 in $\mathscr{D}$. We will show that there exists another distribution $\mathscr{D}'$ over panels whose probability allocation is leximin-fairer than the probability allocation of $\mathscr{D}$, which will contradict the optimality of LexiMin.

Let d denote the probability mass function of $\mathscr{D}$, mapping each possible panel of the instance to the probability with which it is returned in $\mathscr{D}$. Furthermore, define for each panel P a second panel $swap(P)$, in which i_1 is exchanged for i_2 and vice versa:

$$swap(P) := \begin{cases} P \setminus \{i_1\} \cup \{i_2\} & \text{if } i_1 \in P \text{ and } i_2 \notin P \\ P \setminus \{i_2\} \cup \{i_1\} & \text{if } i_2 \in P \text{ and } i_1 \notin P \\ P & \text{otherwise.} \end{cases}$$

Since i_1 and i_2 have exactly the same features, $swap(P)$ is also a quota-compliant panel.

Now, define $\mathscr{D}_{swap}$ by the probability mass function d_{swap} with values

$$d_{swap}(P) := d(swap(P)).$$

For each agent $i \notin \{i_1, i_2\}$, their selection probability is equal in $\mathscr{D}$ and $\mathscr{D}_{swap}$, because the agent is included in a panel P iff they are included in $swap(P)$. Also, the selection probability of i_1 in $\mathscr{D}_{swap}$ is p_2 and that of i_2 is p_1.

Now define the symmetrization $\mathscr{D}'$ of $\mathscr{D}$ over i_1 and i_2 as the mixture of distributions $\frac{1}{2}\mathscr{D} + \frac{1}{2}\mathscr{D}_{swap}$. In this distribution, each agent $i \notin \{i_1, i_2\}$ is selected with the same probability as in $\mathscr{D}$, but i_1 and i_2 are both selected with probability $(p_1 + p_2)/2$. This probability allocation is leximin-fairer than that of $\mathscr{D}$, contradiction. □

15.4 Proportionality

If a selection algorithm satisfies proportionality, each agent i should, on every instance, receive at least a $1/n$ fraction of the selection probability they would receive under their most preferred probability allocation for this instance (i.e., the probability allocation chosen if i was a dictator[66]). Note that, if i is contained in some panel P, the panel distribution that deterministically outputs P gives rise to a probability allocation in which i is chosen with probability 1. Thus, proportionality requires that i is selected with probability at least $1/n$. Else, if i is not contained in any panel, no probability allocation gives them positive selection probability, and proportionality does not guarantee them any minimum selection probability. Consequently, proportionality in the panel-selection setting can be defined as follows:

Definition 7 (proportionality)**.** A selection algorithm guarantees *proportionality* if, on all instances, each agent i has a selection probability of at least $1/n$ unless they are not contained in any possible panel.

Theorem 8. LEXIMIN *guarantees proportionality.*

Proof. Fix an arbitrary instance. Partition the agents N into two sets: the agents N^+ that are contained in at least one panel and the agents N^- that are not contained in any panel. Since at least one panel must exist, $N^+ \neq \emptyset$.

First, consider the leximin-optimal probability allocation $\vec{p}_{lex}$. Assume for the sake of contradiction that LEXIMIN violates proportionality on this instance, i.e., that some agent in N^+ is selected with probability $p < 1/n$.

Under this assumption, we will construct another panel distribution with a probability allocation $\vec{p}_{alt}$ that is strictly leximin-fairer than $\vec{p}_{lex}$, which will contradict the optimality of $\vec{p}_{lex}$. For each $i \in N^+$, let P_i be a panel such that $i \in P_i$. Then, consider the distribution

over panels resulting from choosing an agent $i \in N^+$ uniformly at random and returning P_i. Call the corresponding probability allocation $\vec{p}_{alt}$. Note that each $i \in N^+$ will be contained in the panel selected in this way with probability at least $1/|N^+| \geq 1/n$.

Clearly, each agent in N^- must receive selection probability 0 in both $\vec{p}_{lex}$ and $\vec{p}_{alt}$. Since the next-lower selection probability of $\vec{p}_{alt}$ is at least $1/n$, and since the next-lower selection probability of $\vec{p}_{lex}$ is $p < 1/n$, $\vec{p}_{alt}$ would be leximin-fairer than $\vec{p}_{lex}$, contradiction. □

References

47. Courant, D. *Sortition and Democratic Principles: A Comparative Analysis* in *Legislature by Lot: Transformative Designs for Deliberative Governance* (Verso, 2019).
48. Rae, D. W. *Equalities* (Harvard University Press, 1989).
49. Mansbridge, J. Should Blacks Represent Blacks and Women Represent Women? A Contingent "Yes". *J. Politics* **61,** 628–657 (1999).
50. Gleixner, A. *et al.* MIPLIB 2017: Data-Driven Compilation of the 6th Mixed-Integer Programming Library. *Math. Program. Comput.* (2021).
51. Dowlen, O. Sorting Out Sortition: A Perspective on the Random Selection of Political Officers. *Political Stud.* **57,** 298–315 (2009).
52. Steel, D., Bolduc, N., Jenei, K. & Burgess, M. Rethinking Representation and Diversity in Deliberative Minipublics. *J. Deliberative Democracy* **16,** 46–57 (2020).
53. Dowlen, O. *The Political Potential of Sortition: A Study of the Random Selection of Citizens for Public Office* (Imprint Academic, 2008).
54. Garey, M. R. & Johnson, D. S. *Computers and Intractability: A Guide to the Theory of NP-Completeness* (Freeman, 1979).
55. Adleman, L. Two Theorems on Random Polynomial Time. *In Proc. IEEE SFCS,* 75–83 (1978).
56. Karp, R. M. & Lipton, R. J. Some Connections between Nonuniform and Uniform Complexity Classes. *In Proc. ACM STOC,* 302–309 (1980).
57. Bradley, S. P., Hax, A. C. & Magnanti, T. L. *Applied Mathematical Programming* (Addison-Wesley, 1977).
58. Gondzio, J., González-Brevis, P. & Munari, P. Large-Scale Optimization with the Primal-Dual Column Generation Method. *Math. Program. Comput.* **8,** 47–82 (2016).
59. Constantino, M., Klimentova, X., Viana, A. & Rais, A. New insights on integer-programming models for the kidney exchange problem. *Eur. J. Oper. Res.* **231,** 57–68 (2013).
60. Dickerson, J. P., Manlove, D. F., Plaut, B., Sandholm, T. & Trimble, J. Position-Indexed Formulations for Kidney Exchange. *In Proc. ACM EC,* 25–42 (2016).

61. Nace, D. & Orlin, J. B. Lexicographically Minimum and Maximum Load Linear Programming Problems. *Oper. Res.* **55,** 182–187 (2007).

62. Schrijver, A. *Theory of Linear and Integer Programming* (Wiley, 1986).

63. Norwegian Centre for Research Data. *European Social Survey Round 8 Data, data file edition 2.1* (2016).

64. Thomson, W. *Introduction to the Theory of Fair Allocation* in *Handbook of Computational Social Choice* 261–283 (Cambridge University Press, 2016).

65. Elkind, E., Faliszewski, P., Skowron, P. & Slinko, A. Properties of Multiwinner Voting Rules. *Soc. Choice Welf.* **48,** 599–632 (2017).

66. Conitzer, V., Freeman, R. & Shah, N. Fair Public Decision Making. *In Proc. ACM EC,* 629–646 (2017).
